# Multi-Hydrogen Bonding on Quaternized-Oligourea Receptor Facilitated Its Interaction with Bacterial Cell Membranes and DNA for Broad-Spectrum Bacteria Killing

**DOI:** 10.3390/molecules29163937

**Published:** 2024-08-21

**Authors:** Xiaojin Yan, Fan Yang, Guanghao Lv, Yuping Qiu, Xiaoying Jia, Qirong Hu, Jia Zhang, Jing Yang, Xiangyuan Ouyang, Lingyan Gao, Chuandong Jia

**Affiliations:** 1 Key Laboratory of Synthetic and Natural Functional Molecule of the Ministry of Education, College of Chemistry and Materials Science, Northwest University, Xi’an 710069, China; 202220978@stumail.nwu.edu.cn (X.Y.); 202321853@stumail.nwu.edu.cn (F.Y.); 202310205@stumail.nwu.edu.cn (G.L.); 202210208@stumail.nwu.edu.cn (Y.Q.); 202210217@stumail.nwu.edu.cn (X.J.); 2020111068@stumail.nwu.edu.cn (Q.H.); ouyangxiangyuan@163.com (X.O.); 2 Shaanxi Key Laboratory of Degradable Biomedical Materials, Shaanxi R&D Center of Biomaterials and Fermentation Engineering, School of Chemical Engineering, Northwest University, Xi’an 710069, China; 202220965@stumail.nwu.edu.cn (J.Z.); yangjing2018@nwu.edu.cn (J.Y.)

**Keywords:** supramolecular chemistry, hydrogen bonds, anion receptor, host–guest systems, DNA cleavage

## Abstract

Herein, we report a new strategy for the design of antibiotic agents based on the electrostatic interaction and hydrogen bonding, highlighting the significance of hydrogen bonding and the increased recognition sites in facilitating the interaction with bacterial cell membranes and DNA. A series of quaternary ammonium functionalized urea-based anion receptors were studied. While the monodentate mono-urea **M1**, bisurea **M2**, and trisurea **M3** failed to break through the cell membrane barrier and thus could not kill bacteria, the extended bidentate dimers **D1**–**D3** presented gradually increased membrane penetrating capabilities, DNA conformation perturbation abilities, and broad-spectrum antibacterial activities against *E. coli*, *P. aeruginosa*, *S. aureus*, *E. faecalis*, and *S. epidermidis*.

## 1. Introduction

Currently, the emergence of new antibiotics lags far behind the rise in antibiotic resistance, which has become one of the most pressing issues in global public health [1,2,3]. In their ongoing war against antibiotics, bacteria are continually evolving to acquire resistance, by decreasing cell permeability, changing the structure of antibiotic targets, or directly inactivating antibiotics [4]. The search for new antibiotic agents with novel structures and mechanisms is urgently required [5,6]. Given the fact that organophosphate anions are ubiquitous in cell membrane phospholipids and DNA, nature has evolved cationic host-defense peptides to kill bacteria by targeting and consequentially disintegrating these negatively charged biomolecules through electrostatic interactions [7,8,9]. This strategy has inspired the prosperous development of various cationic antibiotic agents, including cationic polymers [10,11,12,13], ionic liquids [14,15,16], metal nanomaterials [17,18], and supramolecular assemblies [19,20,21].

In biological systems, both hydrogen bonding and electrostatic interactions are critically involved in the recognition of organophosphate anions by proteins [22], while hydrogen bonding has received much less attention than electrostatic interactions in the design of antibiotic agents [23,24,25,26,27,28,29]. Pioneering studies in supramolecular chemistry have disclosed the bioactivities of hydrogen-bonding anion receptors in transmembrane transport [29,30,31] and DNA condensation [32,33]; it is rational to assume that positively charged, hydrogen-bonding anion receptors might be a promising class of antibacterial agents, which combine the bioactivities of electrostatic interactions and hydrogen bonding [34,35,36]. However, only a very few hydrogen-bonding-based anion receptors can bind phosphate-derivated anions in water because of the challenge of high dehydration energies [37,38,39,40,41,42,43,44,45]. In our efforts to develop hydrogen-bonding-based anion receptors based on *ortho*-phenylene oligourea [46,47,48,49], we recently realized selective binding of ATP in water by a quaternized trisurea [47]. More importantly, urea is often utilized for destabilizing the helical and folded conformations of nucleic acids by forming stacking interactions and hydrogen bonds [19,50]. Hence, we hypothesized that quaternized, urea-based anion receptors might present as a new type of antibiotic that target the phosphate-derivated anions.

With this in mind, we synthesized three anion receptors, **D1**–**D3**, each equipped with a double-positively charged quaternary ammonium headgroup and a different number of urea groups, including a mono-urea **M1**, a bisurea **M2**, and a trisurea **M3** (Scheme 1, Figures S1–S24). The quaternary ammonium is a well-known membrane disruptor widely utilized in the design of cationic antibiotic agents, and it is also necessary to overcome the usually poor water solubility of hydrogen-bonding receptors and enhance their anion binding affinity [40,43,44,47]. The ortho-phenylene-connected oligourea is one of the most extensively studied hydrogen-bonding receptors for phosphate recognition [40,43,46,47,51,52,53,54,55,56].

## 2. Results and Discussion

**Scheme 1:**
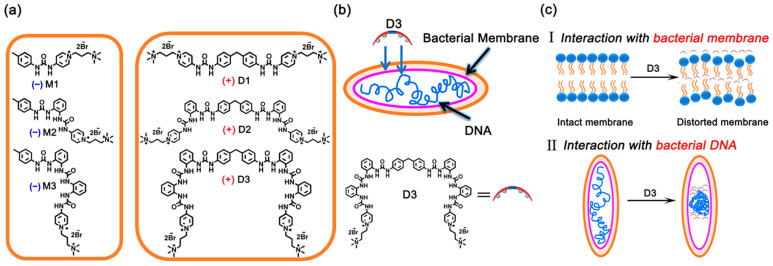
(**a**) Quaternized-oligourea monomers (**M1**–**M3**) and dimers (**D1**–**D3**). (**b**,**c**) The possible pathways of bacteria killing based on the interaction between **D3** and DNA/bacteria member (+ denotes active, − denotes non-active).

**Figure 1:**
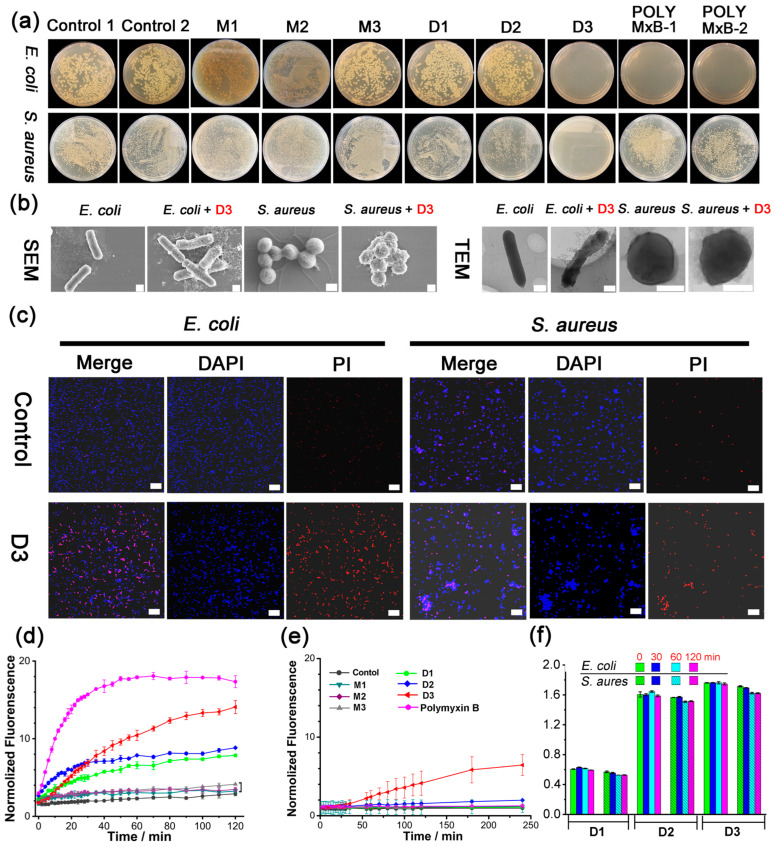
(**a**) Images of *E. coli* and *S. aureus* colonies on the Luria-Bertani (LB) agar plates: untreated (control 1 (PBS), control 2 (DMSO/PBS, 5:95, *v*/*v*)); treated with 30 μM **M1**–**M3**, **D1**–**D3**, Polymyxin B (dissolved in PBS (POLY MxB-1); and dissolved in DMSO/PBS (5:95, *v*/*v*) (POLY MxB-2), respectively. (**b**) SEM (scale bar: 400 nm) and TEM (scale bar: 400 nm) images of untreated and **D3**-treated *E. coli* and *S. aureus*. (**c**) CLSM images of *E. coli* and *S. aureus* after treatment with control (PBS), and urea-based receptor **D3**, respectively. From left to right, the images in columns show merged images, blue signals from DAPI, and red signals from PI. (Scale bar: 20 μm). (**d**,**e**) Cell membrane permeability of (**d**) *E. coli* and (**e**) treated with various receptors (30 μM), using propidium iodide (PI) as the dye (λ_em_ = 617 nm). (**f**) DNA release from *E. coli* and *S. aureus* incubated with **D1**–**D3** (30 μM). PBS (pH = 7.4) containing 5% (*v*/*v*%) DMSO was used as the solvent to ensure the complete resolution of tested compounds in all assays. Data are presented as the mean ± SD, *n* = 3.

The antibacterial activities of **M1**–**M3** against Gram-negative (*E. coli*) and Gram-positive (*S. aureus*) bacteria were evaluated. Unfortunately, none of them presented activities (minimum inhibitory concentration (MIC) > 1.0 mM, Table S1 and Figure 1a). It has been reported in our previous work that the urea-based receptor demonstrated enhanced anion affinities with increased urea units and *ortho*-phenylene oligourea can selectively bind phosphate or ATP in water by a quaternized trisurea [47,53]. We thus extended the monodentate **M1**–**M3** (monomer) to bidentate dimers **D1**–**D3** (dimer) with the 4,4′-methylenebis (phenyl) linker (Scheme 1). Encouragingly, all three dimers **D1**–**D3** displayed broad-spectrum antibacterial activities, with the MICs (in μM) against *E. coli*/*S. aureus* 120/60, 30/30, and 15/15, respectively (Table 1 and Figure 1a). **D1**–**D3** also demonstrated antibacterial activities against *Pseudomonas aeruginosa* (*P. aeruginosa*)*, Enterococcus faecalis* (*E. faecalis*), and *Staphylococcus epidermidis* (*S. epidermidis*), indicating that **D1**–**D3** possessed broad-spectrum antibacterial abilities (Table 1). Moreover, biocompatibility assays revealed that **D1**–**D3** demonstrated much lower cell toxicity for mammalian cells than bacteria at the same concentration, that over 90% of cells were alive at the concentrations below 15 μM, and about 80% of cells survived at 30 μM (Figure S29). In control experiments, two model compounds, **MD1** (Scheme S7, with amino bond instead of urea group) and **MMD1** (Scheme S8, the two hydrogen atoms in the amino bond of **MD1** were all methylated), without urea groups exhibited much worse antibacterial activities against *E. coli*, *P. aeruginosa*, *S. aureus*, *E. faecalis*, and *S. epidermidis* (MIC and MBC results were summarized in Table S2), which confirmed the importance of hydrogen bonding in the antibacterial activities of **D1**–**D3**. Meanwhile, the antibacterial activities of **D1A**–**D3A**, the neutral precursors of **D1**–**D3** with no function groups of the quaternary ammonium headgroups (Table S1), further implied quaternary ammonium salt also contributed to the antibacterial behavior to some extent.

Dynamic light scattering (DLS), scanning electron microscopy (SEM), transmission electron microscopy (TEM), and confocal laser scanning microscopy (CLSM) measurements were carried out to probe the possible antibacterial mechanism of the quaternized-oligourea dimer. Larger aggregates could be clearly observed from the DLS results when the bacterial suspensions of *E. coli* and *S. aureus* were incubated with **D3** for 60 min (Figure S30), indicating the quaternized-oligourea dimer **D3** could interact with the bacterial cells via noncovalent interactions and tends to gather on the cell surface. Then, scanning electronic microscopy (SEM) and transmission electron microscopy (TEM) were employed to investigate the surface morphology of *E. coli* and *S. aureus* treated with **D3**. In contrast with the untreated bacteria’s complete morphology and full cellular content, **D3**-treated bacteria displayed distorted morphology (Figure 1b). Furthermore, the effects of the quaternized-oligourea dimer against bacterial cell viability were confirmed by the CLSM study. After treatment with **D3** for 12 h, increased red fluorescence intensity from propidium iodide (PI) was noted both in *E. coli* and *S. aureus* suspensions, as expected in comparison with the bacteria treated with PBS, indicating that their cell membranes became incomplete (Figure 1c) [57,58,59]. Meanwhile, in bacteria incubated with the same concentration of **D1** and **D2**, less red fluorescence intensities could be observed (Figure S31), suggesting the lower antibacterial activities of **D1** and **D2**, which are in accordance with the MIC results listed in Table 1. 

Subsequently, PI uptake assay and cell membrane integrity assay were carried out to identify the bacterial cell membrane permeability after the treatment with a quaternized-oligourea dimer (Figure 1d,e) [57,58,59]. Treatment of *E. coli* with **M1**–**M3** (1.0 mM) resulted in negligible perturbation of the fluorescence, implying these receptors could not break through the cell membrane barrier and thus excluded the binding with organophosphate anions. On the contrary, 30 μM **D1**–**D3** displayed dramatic membrane permeability activity towards *E. coli* (Figure 1d and Figure S32). Meanwhile, it was found that the enhanced membrane permeability induced by **D1**–**D3** is not big enough to allow the leakage of cytoplasmic nucleic acids from the bacterial cells, because their characteristic absorbance at 260 nm showed no significant changes over 120 min (Figure 1f). This is consistent with the results of the surface morphology changes, in which **D3**-treated bacteria showed a deformed but not damaged morphology. Therefore, the quaternized-oligourea dimer could associate with bacterial cell membranes through noncovalent interactions and further disturb the membrane integrity, finally leading to the cell death.

**Figure 2:**
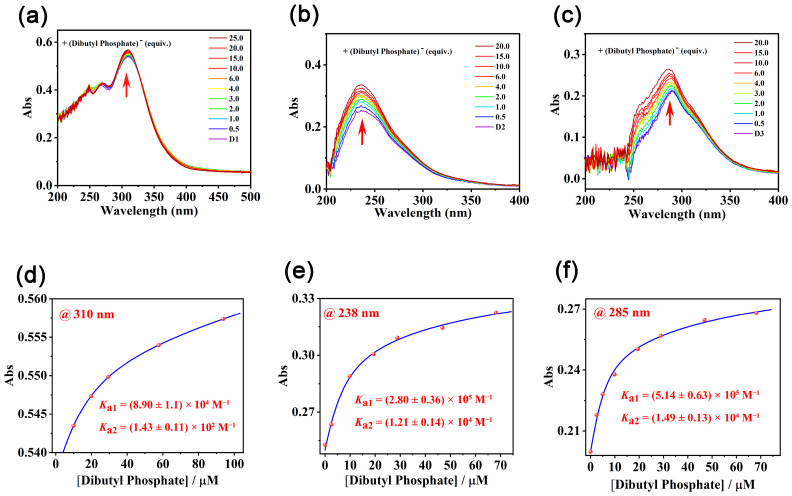
(**a**–**f**) UV-vis absorption spectra of dibutyl phosphate upon titration with **D1**–**D3** in chloroform/DMSO (the DMSO content was 1%). (**d**) The fitted titration profile by following the shifts of UV absorption at 310 nm of **D1**. (**e**) The fitted titration profile by following the shifts of UV absorption at 238 nm of **D2**. (**f**) The fitted titration profile by following the shifts of UV absorption at 285 nm of **D3**.

As **M1**–**M3** and **D1**–**D3** with increased anion-binding sites of urea groups exhibited dramatically different antibacterial activities, efforts were devoted to rationalizing the gradually increased membrane penetrating capabilities of **D1**–**D3** by their binding constants with a model organophosphate anion, dibutyl phosphate, in a nonpolar solvent chloroform. Job’s plot based on UV titrations revealed that all three receptors **D1**–**D3** formed a 1:2 complex with the dibutyl phosphate (Figure S33). The binding affinities of **D1**–**D3** with dibutyl phosphate gradually increased along with the increased urea units on the framework (Figure 2). Meanwhile, the model compound **M1**–**M3**, which could form a complex with dibutyl phosphate in 1:1 stoichiometry (Figure S34g–i), respectively, exhibited much lower binding affinities with the dibutyl phosphate (Table 2 and Figure S34a–f). Apparently, the increased hydrogen-binding sites led to enhanced binding with the organophosphate anion and finally resulted in increased membrane penetrating capabilities.

It has been known that urea can influence the conformation of DNA by forming stacking interactions and multiple hydrogen bonds with nucleic acid bases. As the cell permeability assays have revealed that small molecules can across the compromised cell membrane in the presence of quaternized-oligourea dimers **D1**–**D3**, we hypothesized that **D1**–**D3** can further diffuse into the cell and bind with bacterial DNA to cause the DNA configuration change, which may also lead to cell death. On this basis, the interactions between DNA and **D1**–**D3** were investigated by ^1^H NMR, UV-vis titrations, and atomic force microscopy (AFM) measurement.

Considering the complicated structure of DNA in the ^1^H NMR spectrum, deoxyribonucleic acid sodium salt (DA) and adenosine monophosphate (AMP) were selected as a simplified DNA backbone and model nucleic acid base to investigate the interactions between DNA and **D3**. From the NMR spectra, it could be seen that the peaks of AMP demonstrated upfield shifts, indicating the interactions between the nucleic acid base and **D3** (Figure S36). The addition of **D3** into the solution of DA generated a white flocculent precipitate, indicating rapid interactions occurred between DA and **D3**. The remaining mixture of DA and **D3** was difficult to identify in the NMR spectrum, as the peaks of DA were too broad to see the change after the addition of **D3** (Figure S35). These results gave strong evidence that **D3** could bind with DNA components through noncovalent interactions.

**Figure 3:**
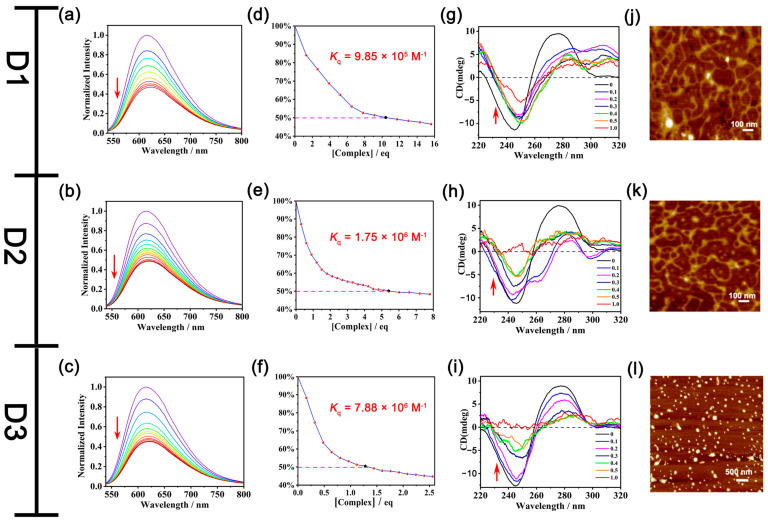
(**a**) Fluorescence spectra of the DNA-EB complex (λ_ex_ = 520 nm, λ_em_ = 610 nm) in the presence of (**a**) **D1**, (**b**) **D2**, and (**c**) **D3**, respectively (each titration profile is labeled as a unique color and the red arrows denote the profile changes following increasing titrations). [C_CT-DNA_] = 0.45 mM, [C_EB_] = 1.3 μM. The concentrations of **D1**, **D2**, and **D3** in the titration curves can be found in the experimental section of “DNA binding study”. *K*_q_ of **D1** (**d**), **D2** (**e**), and **D3** (**f**) with CT-DNA, respectively. Taking figure (**d**) as an example, [complex] = [C_D1_] × V_D1_/[[C_EB_] × (V_EB_ + V_D1_)], [C_EB_] = 1.3 μM, V_EB_ = 3000 μL. CD spectra of CT-DNA (0.1 mM) with the addition of different molar ratios of **D1** (**g**), **D2** (**h**), and **D3** (**i**), respectively. AFM images of pDNA (**g**) before and after the addition of **D1** (**j**), **D2** (**k**), and **D3** (**l**), respectively, with a concentration of 15 μM of **D1** and **D2** and 7.5 μM of **D3**.

Then, a competitive ethidium bromide (EB) displacement assay was performed to decipher the binding mode of DNA with quaternized urea-based receptors [60,61,62,63]. From the fluorescence spectra of the DNA-EB system, along with the addition of **D3**, the emission intensity of the EB-DNA system at 610 nm decreased significantly upon the increased concentration of **D3** (Figure 3c), indicating that **D3** behaved as an intercalator, which could replace the DNA-bound EB. From the titration profile, *K*_q_ of **D3** with DNA is calculated as 7.88 × 10^6^ M^−1^ (Figure 3f). As anticipated, **D1** and **D2** were also found to bind with DNA in a similar manner as **D3**, and the *K*_q_ of **D1** (9.85 × 10^5^ M^−1^, Figure 3d) and **D2** (1.75 × 10^6^ M^−1^, Figure 3e) showed lower binding propensities than **D3**. Monomers **M1**–**M3** gave EB-quenching constants 2–4 orders of magnitude lower than the corresponding dimers **D1**–**D3** (Table S3 and Figure S37), indicating the importance of the increased urea units in DNA binding. These results suggest that **D1**–**D3** can behave as intercalators in the interaction with DNA, and the intercalation is enhanced by increased hydrogen binding sites.

Circular dichroism (CD) spectroscopy was also carried out to further observe the global changes in the DNA conformation induced by **D3** [60,61,62,63]. Intrinsic CT-DNA showed a positive band at 275 nm ascribed to the base stacking and a negative band at 245 nm due to the helicity of DNA (Figure 3i), which is the signature peak of B-DNA in the CD spectrum. With the addition of **D3**, the CD values of 275 nm and 245 nm bands of CT-DNA were gradually decreased, suggesting **D3** could perturb the base stacking and helicity bands, leading to the destabilization of the right-handed B form of CT-DNA. When the molar ratio of **D3** to CT-DNA progressively increased to 0.5, the ellipticity of the 245 nm band decreased by 64%, with a slight red shift of 5 nm, while the ellipticity of the 275 nm band decreased by 78% without any significant shift. The characteristic bands of CT-DNA disappeared with the addition of **D3**, with the mole ratio reaching 1. The change of the positive band and negative band of CT-DNA reflected that **D3** could influence the secondary structure of DNA by perturbing its base stacking and helicity.

Similar changes to CT-DNA in the CD spectra could also be observed in the presence of **D1** and **D2**. The ellipticity of the 275 nm band and the 245 nm band both exhibited a decreasing tendency with the addition of **D1** and **D2**. With a 0.5 molar ratio of **D1**, the ellipticity of the 245 nm and 275 nm bands decreased by 17% and 61%, respectively. The intensity at the 245 nm band further decreased to 53.7% and the band at 275 nm disappeared in the presence of a ratio of 1 equivalent **D1** (Figure 3g). While with a 0.5 equivalent of **D2**, there was a 64% and 44.2% decrease of the intensity at 245 nm and 275 nm, respectively. The characteristic bands of CT-DNA disappeared when the molar ratio of **D2** reached 1 (Figure 3h).

However, quaternized-oligourea monomer **M3** only exerted a small influence on the DNA conformation, and the ellipticity of the 275 nm band and 245 nm band only decreased by 13% and 6.9%, respectively (Figure S38). This indicated the bridging diphenylmethane also contributed to the intercalation of quaternized-oligourea dimers into the DNA secondary structure, leading to the conformation change of DNA. In general, the major feature of the CD spectra is the decrease in the ellipticity for both bands of CT-DNA in the presence of quaternized-oligourea dimers **D1**–**D3**, and their perturbation on the base-stacking and helicity bands of CT-DNA follows the order **M3** < **D1** < **D2** < **D3**.

In order to further visualize the effect of **D3** on DNA, AFM measurements were performed. pBR322 Plasmid DNA (pDNA), the most commonly used *E. coli* cloning vector, was used in the AFM study. It was noted that the intrinsic dispersive supercoiled structures of pDNA changed into small particles with the incubation of **D3** for 30 min (Figure 3l), illustrating that **D3** could interact with pDNA and further influence the morphology of pDNA through noncovalent interactions. While for the pDNA treated with **D1** and **D2** with a higher concentration (15 μM), no obvious morphological change of the pDNA could be observed (Figure 3j,k, Figures S39 and S40). Only with much higher concentrations could a significant DNA morphology change be noted from AFM (Figure S40).

The DNA binding capability of **D3** in vitro and in vivo was further evaluated by agarose gel electrophoresis (Figure S41). **D3**/pDNA complexes at different concentrations of **D3** ranging from 1.9 to 60 μM under 30 min culture were electrophoresed in agarose gel, where naked pDNA was used as a reference. **D3** could partially retard the migration of pDNA in agarose gel over 3.75 μM (Figure S41a), which might be ascribed to the conformation and surface charge change of the **D3**/pDNA complex in agarose gel electrophoresis, as indicated by AFM results of pDNA incubated with **D3**. Fully retarding behavior was noted when the concentration of **D3** reached up to15 μM (Figure S41a). Subsequently, the binding of **D3** with DNA molecules in *E. coli* cells was also assessed. After the bacterial suspension was cocultured with **D3** for 12 h, DNA molecules were extracted and studied by agarose gel electrophoresis. In contrast to the DNA in the agarose gel treated with PBS, the migration of DNA was partially retarded in agarose gel in the presence of **D3** with a concentration of 3.75 μM, and no DNA migration occurred when the concentration of **D3** was higher (Figure S41b). From these results, we considered that **D3** may diffuse into the cell and bind with DNA via noncovalent interactions, leading to the configuration change of DNA and causing cell death.

Taken together, we postulate that quaternized-oligourea dimers have a multimodal bactericidal mechanism. Initially, quaternized-oligourea dimers bind with phosphate-derivated anions on bacterial cell membranes through noncovalent interactions, increasing the cell membrane permeability. Meanwhile, they can further diffuse into the cell through the perturbated cell membrane and bind with DNA molecules, resulting in the conformation change of DNA and cell death.

## 3. Experimental Section

### 3.1. Materials

All chemicals were purchased from Sigma (Darmstadt, Germany); unless stated otherwise. Solvents were either employed as purchased or dried before use by the usual laboratory methods. The ^1^H and ^13^C NMR spectra were recorded on Bruker AVANCE III-400 MHz spectrometers (Rheinstetten, Germany). The ESI-MS spectra were acquired using a Bruker micrOTOF-Q II ESI-TOF-MS LC/MS/MS spectrometer (Bremen, Germany). Transmission electron microscopic investigations were carried out on a FEI Talos F200X (Thermo Fisher Scientific, Eindhoven, The Netherlands). Scanning electron microscopic investigations were carried out on a HITACHI SU8010 (Hitachinaka, Japan). Atomic force microscopic investigations were carried out on a BRUKER Multimode 8 (Santa Barbara, CA, USA). Fluorescence spectra were performed by using a Horiba Fluorolog-3 spectrometer (Kyoto, Japan).

Bacterial strains *E. coli* (ATCC 25922), *S. aureus* (ATCC 25923) bacteria, *E. faecalis* (ATCC 29212), *P. aeruginosa* (ATCC 27853), and *S. epidermidis* (ATCC 12228) were adopted in this study. The bacteria were initially streaked from −80 °C glycerol stocks on lysogeny broth (LB). The L929 cell line used in our manuscript was obtained from Shanghai Fuheng Biotechnology Co., Ltd. (Shanghai, China). After growth on LB agar plates, the cells were cultured from a fresh single colony in LB. All experiments were conducted at 37 °C. All glassware used in this study was sterilized before testing. 

### 3.2. Determination of MIC and MBC Values

Two-fold serial dilutions of the test compounds and polymyxin B were prepared in a microdilution plate. Strains of *E. coli* and *S. aureus* were used in this test. An overnight culture was diluted in an LB medium to obtain a bacterial concentration of 10^6^ CFU/mL. The inoculum was dispensed into the microdilution plate with the serially diluted test compounds and the microdilution plate incubated. The microdilution plate was read to determine the MIC value. Then, a portion of each well was plated on the appropriate agar media, the agar incubated, and the colonies checked to determine the MBC values.

### 3.3. Cell Viability Assay

The cytotoxicities of **D1**, **D2**, and **D3** were evaluated by Cell Counting Kit-8 (CCK8, Tansoole) (Shanghai, China) assay. L929 cells were seeded into 96-well plates at a concentration of 10^4^ cells per well. After 24 h incubation, the culture medium was removed and replaced with 100 μL of fresh medium containing PBS, **D1**, **D2**, and **D3**, respectively. The cells were incubated for another 24 h. Then, each well was replaced by 100 μL of fresh medium added with 10 μL of CCK-8 reagent. After 2 h of incubation, the optical density was measured at 450 nm by a spectrophotometer. The percentage of cell viability was calculated using the following equation:

Cell viability% = (*A*_450, treated-x h_ − *A*_450, blank_)/(*A*_450, treated-0 h_ − *A*_450, blank_) × 100%,

where *A*_450, treated-x h_ is the absorbance in the presence of tested compounds at a certain timepoint (24 h), *A*_450, treated-0 h_ is the absorbance in the presence of tested compounds at 0 h, and *A*_450, blank_ is the absorbance in the presence of PBS, respectively.

### 3.4. DLS Study

**D3** was dissolved in DMSO/PBS (5%, *v*/*v*, DMSO was added in the cases of **D3** to ensure good solubility). **D3** (30 μM, 100 μL) was added into *E. coil* or *S. aureus* suspensions (10^6^ CFU/mL, 100 μL). The aggregate sizes of the bacterial suspensions with or without **D3** were monitored for 60 min.

### 3.5. Scanning Electron Microscopy (SEM) Study

The strain of *E. coli* and *S. aureus* was used in this test. An overnight culture medium was diluted in LB to obtain the bacterial concentration of 10^6^ CFU/mL, and added into a 96-well plate with 100 μL bacterial suspension and 100 μL tested compounds. The 96-well plate was incubated at 37 °C for 24 h, respectively. Then, the bacterial suspensions were centrifuged (3000 rpm, 10 min) to remove the supernatant. The precipitate was resuspended in PBS (pH = 7.4) to repeat the previous centrifugation process two times. Glutaraldehyde (2.5%) was added to the bacterial suspension and the mixture was incubated for 4 h. The mixture was then centrifuged and washed with PBS (pH = 7.4) three times. The sample was sequential dehydrated by different volumes (30%, 50%, 70%, 80%, 90%, and 100%) of ethanol for 10 min and further incubated with 100% ethanol for 1 h. The sample was finally dropped onto the silicon wafer and dried for scanning electron microscopy (SEM) study.

### 3.6. Transmission Electron Microscopy (TEM) Study

Bacterial strains of *E. coli* and *S. aureus* were used in this test. An overnight culture medium was diluted in LB to obtain the bacterial concentration of 10^6^ CFU/mL, and added into a 96-well plate with 100 μL bacterial suspension and 100 μL tested compounds. The 96-well plate was incubated at 37 °C for 24 h, respectively. After treatment, the bacterial suspensions were centrifuged at 10,000 rpm for 10 min and washed carefully with 0.1 M PBS. Afterwards, pellets were fixed with 0.5 mL of 2.5% gluteraldehyde solution, and then subjected to a series of alcohol dehydration process (30%, 50%, 70%) followed by PBS washing. The pellets were dispersed in 1 mL of pure ethanol (100%), mixed with aqueous sodium phosphowolframate solution (2%) and incubated for 30 min. Finally, the sample was dropped onto the carbon-filmed copper grids and dried for transmission electron microscopy (TEM) study.

### 3.7. Confocal Laser Scanning Microscopy (CLSM) Study

Strains of *E. coli* and *S. aureus* were used in this test. An overnight culture was diluted in LB to obtain a bacterial concentration of 10^6^ CFU/mL and added into a 96-well plate with 100 μL bacterial suspension and 100 μL tested compounds (15 μM). The 96-well plate was incubated at 37 °C for 12 h. Two different dyes, DAPI and PI, were used. The bacterial suspensions were centrifugated (10,000 rpm, 10 min) and further incubated with PI (5 mg/L) and DAPI (5 mg/L) for 15 min in the dark. Then, the suspensions were centrifugated (10,000 rpm, 5 min). Excess PI and DAPI were removed and washed three times with PBS. Then, bacterial strains were resuspended in PBS and pipetted into 15 mm glass bottom dishes and observed by confocal laser scanning microscopy (CLSM). The images were taken using a confocal laser scanning platform (Nikon A1, Tokyo, Japan).

### 3.8. Propidium Iodide (PI) Uptake Assay

Mid-log phase (grown for 6 h) bacterial cells (*E. coli*, *S. aureus*) were harvested (4000 pm, 4 °C, 10 min), washed, and resuspended in a PBS buffer of pH 7.4. Then, test compounds (30 μM, 20 μL) were added to a cuvette containing 160 μL (OD_600_ = 0.30) of bacterial solution and 20 μL (10 μM) of propidium iodide (PI). The fluorescence of the track was monitored for 120 min using a Thermo scientific Varioskan Flash spectrofluorometer (Thermo Fisher, Waltham, MA, USA) at an excitation wavelength of 535 nm and an emission wavelength of 617 nm. The uptake of PI was measured by the increase in fluorescence of PI for 120 min as a measure of inner membrane permeabilization.

### 3.9. DNA Release Study

*E. coli* and *S. aureus* suspensions (OD_600_ = 0.30) were prepared and tested for cell membrane integrity. A bacterial suspension of 100 μL was transferred to wells in a centrifuge tube. Tested compound **D1** (or (**D2**), (**D3**)) (30 μM) was added to *E. coli* and *S. aureus* suspension wells, respectively, and co-cultured in batches at 37 °C for 0, 30, 60, and 120 min. Then, the bacterial suspensions were centrifugated (10,000 rpm, 10 min) to remove the supernatant, and the absorbance was measured at 260 nm. Data are presented as the mean ± SD, n = 3 (Shimadzu UV-3600 Plus) (Shimane Prefecture, Japan).

### 3.10. Determination of the Binding Constants by UV-Vis Titrations

A 30 μM stock solution of **M1** (or a 20 μM stock solution of **M2** (or **M3**), a 10 μM stock solution of **D1**, and a 5 μM stock solution of **D2** (or **D3**)) was prepared in DMSO/CHCl_3_ (1%, *v*/*v*, DMSO was added in the cases of **M1**–**M3** and **D1**–**D3** to ensure a good solubility), which was used as a solvent to prepare 4.5 mM stock solutions of tested Dibutyl Phosphate as the titrants. This keeps the concentration of **M1** constant, thus avoiding the dilution effect. To 3.0 mL of the stock solution of **M1**–**M3** and **D1**–**D3** in a 1 cm × 1 cm quartz cell, small portions of the titrants were added gradually to obtain satisfied titration profiles. The association constants (*K*_a_) were determined by fitting the titration profiles with the Dynafit program (dynafit 4) [64].

### 3.11. DNA Binding Study

The apparent binding constants (quenching constants *K*_q_) of **M1**–**M3** and **D1**–**D3** with CT-DNA were calculated according to the literature [60,61,62,63], by displacement of the fluorescent dye ethidium bromide (EB). Stock solutions of **M1**–**M3** (10 mM), **D1**, **D2**, and **D3** (10, 2.5, and 1.25 mM) in the buffer (pH = 7.26, 5 mM Tris-HCl, 50 mM NaCl) were titrated to the EB-DNA in the same buffer, and the fluorescence was tracked (λ_ex_ = 520 nm, λ_em_ = 610 nm for EB-DNA). The quenching constants (*K*_q_) were calculated using the equation

*K*_EB_ [EB] = *K*_q_ [receptor]

[EB] = 1.3 μM, [receptor] is the final concentration at which there is a 50% quenching of the fluorescence. *K*_EB_ was reported as 1.0 × 10^7^ M^−1^.

In the titration experiments of Figure 2a–c, the concentration of **D1** in Figure 2a was 0, 1.69 μM, 3.38 μM, 5.07 μM, 6.76 μM, 8.44 μM, 10.10 μM, 11.80 μM, 13.50 μM, 15.20 μM, 16.90 μM, 18.50 μM, and 20.20 μM, respectively. The concentration of **D2** in Figure 2b was 0, 0.39 μM, 0.78 μM, 1.17 μM, 1.56 μM, 1.95 μM, 2.34 μM, 2.73 μM, 3.12 μM, 3.51 μM, 3.89 μM, 4.28 μM, 4.67 μM, 5.06 μM, 5.45 μM, 5.84 μM, 6.22 μM, 6.61 μM, 7.00 μM, 7.39 μM, 7.78 μM, 8.16 μM, 8.55 μM, 8.94 μM, 9.33 μM, 9.71 μM, and 10.1 μM, respectively. The concentration of **D3** in Figure 2c was 0, 0.21 μM, 0.42 μM, 0.62 μM, 0.83 μM, 1.04 μM, 1.25 μM, 1.45 μM, 1.66 μM, 1.87 μM, 2.08 μM, 2.28 μM, 2.49 μM, 2.70 μM, 2.91 μM, 3.11 μM, and 3.32 μM, respectively.

### 3.12. CD Experiments

A reserve solution of 0.1 mM CT-DNA was prepared with buffer (5 mM Tris-HCl, 50 mM NaCl, pH = 7.26), and the concentration of CT-DNA was determined by UV absorption at 260 nm, with 6600 M^−1^ cm^−1^ as the molar absorption coefficient. The ratio of UV absorption rates (A_260_/A_280_) at 260 and 280 nm is approximately 1.8, indicating that the DNA solution is sufficiently free of protein. **M3** and **D1**–**D3** (10 mM) was gradually added to CT-DNA solution (0.1 mM), respectively. CD spectra of these three experiments were recorded at room temperature.

### 3.13. Bacterial DNA Extraction Process

The bacterial DNA was extracted by a TIANamp Bacteria DNA Kit (TIANGE BIOTECH, Beijing, China). The following were provided by the Kit: buffers GB, GD, and TE, proteinase K, and spin columns. A bacterial suspension of *E. coli* was incubated with or without tested compounds for 12 h (2 mL, OD_600_ = 1.0). The sample was centrifuged at 10,000 rpm for 1 min to remove the supernatant. A quantity of 110 μL of STE buffer (50 mM NaCl, 10 mM Tris-HCl, 0.5 mM EDTA) and 70 μL of Lysozyme (50 mg/mL) solution was added to the precipitate and culture at 37 °C for 30 min. Then, 20 μL of proteinase K solution was introduced to the mixture. A quantity of 220 μL buffer GB was further added and left for 10 min at 70 °C. Subsequently, 220 μL of anhydrous ethanol was added to the solution and oscillated for 15 s. The resulting sample was transferred into a spin column and centrifuged at 12,000 rpm for 30 s to remove the supernatant. A quantity of 500 μL of buffer GD was added to spin columns and centrifuged at 12,000 rpm for 30 s to remove the supernatant. A quantity of 600 μL of buffer PW was added to the spin columns and centrifuged at 12,000 rpm for 30 s to remove the supernatant. Finally, 100 μL of buffer TE was added to the spin columns. The solution was kept for 5 min at room temperature and then centrifuged at 12,000 rpm for 120 s to afford DNA.

### 3.14. Agarose Gel Electrophoresis

Gel electrophoresis was run on a 1% (*w*/*v*) agarose gel at 60 V for 30 min. Then, the gel was immersed in EB solution (1.2 μg/mL) for 15 min and photographed by means of a UV transilluminator and WD-9415B gel documentation system 5 (OI 100Touch) (Beijing Liuyi Biotechnology, Beijing, China). The light irradiation was performed using an Xe lamp (CEL-HXF300 14V 50W) (Beijing Zhongjiao Jinyuan Technology Co., Ltd., Beijing, China) with an optical filter which provides visible light at around 450 nm. The light intensity was around 50 mW cm^−2^, and the distance was kept at 10 cm.

## 4. Conclusions

In conclusion, we have developed a novel type of broad-spectrum antibacterial agent based on quaternary ammonium functionalized urea-based anion receptors that combines the bioactivities of electrostatic interaction and hydrogen bonding that arise from targeting the organophosphate anions in the cell membrane and DNA.

## Figures and Tables

**Table 1:** In vitro antibacterial activities of compounds **D1**–**D3**.

	Bacterial Strains									
	*E. coli*		*P. aeruginosa*		*S. aureus*		*S. epidermidis*		*E. faecalis*	
	MIC	MBC	MIC	MBC	MIC	MBC	MIC	MBC	MIC	MBC
**D1**	120	240	60	120	60	120	1.88	3.75	1.88	3.75
**D2**	30	60	30	60	30	60	1.88	3.75	0.94	1.88
**D3**	15	30	15	30	15	30	1.88	3.75	0.47	0.94
Polymyxin B	1.88	3.75	-	-	60	120	-	-	-	-

MIC is the minimum inhibitory concentration, and MBC is the minimum bactericidal concentration. MIC and MBC in μM (- not detected).

**Table 2:** The association constants (*K*_a_) of **M1**–**M3** and **D1**–**D3** with dibutyl phosphate.

Compound	K_a_/M^−1^	Compound	K_a1_/M^−1^	K_a2_/M^−1^
**M1**	(7.08 ± 0.64) × 10^3^	**D1**	(8.90 ± 1.1) × 10^4^	(1.43 ± 0.11) × 10^2^
**M2**	(1.88 ± 0.1) × 10^4^	**D2**	(2.80 ± 0.36) × 10^5^	(1.21 ± 0.14) × 10^4^
**M3**	(2.71 ± 0.19) × 10^4^	**D3**	(5.14 ± 0.63) × 10^5^	(1.49 ± 0.13) × 10^4^

## Data Availability

Data are contained within the article and Supplementary Materials

## Supplementary Materials

The following supporting information can be downloaded at: https://www.mdpi.com/article/10.3390/molecules29163937/s1, including the synthesis of ligands, additional data of bacteria killing studies.
