# Supplementary material for: Multi-Hydrogen Bonding on Quaternized-Oligourea Receptor Facilitated Its Interaction with Bacterial Cell Membranes and DNA for Broad-Spectrum Bacteria Killing
Source: Molecules. 2024 Aug 21;29(16):3937. doi: 10.3390/molecules29163937 (PMC11357520; doi:10.3390/molecules29163937)
Supplement: Supplementary file 1 [file molecules-29-03937-s001.zip › molecules-3158192-supplementary.pdf]

Supporting Information

Multi-Hydrogen Bonding on Quaternized-Oligourea Receptor Facilitated Its Interaction with Bacterial Cell Membranes and DNA for Broad-Spectrum Bacteria Killing

Xiaojin Yan [1,†], Fan Yang [1,†], Guanghao Lv [1], Yuping Qiu [1], Xiaoying Jia [1], Qirong Hu [1], Jia Zhang [2], Jing Yang [2], Xiangyuan Ouyang [1], Lingyan Gao [1,*] and Chuandong Jia [1,*]

* Correspondence: gaolingyan@nwu.edu.cn (L.G.); jcd2015@nwu.edu.cn (C.J.)

Table of Contents

1. Synthesis

2. Bacteria Killing Studies

3. Reference

1. Synthesis

1.1. Synthesis of M1

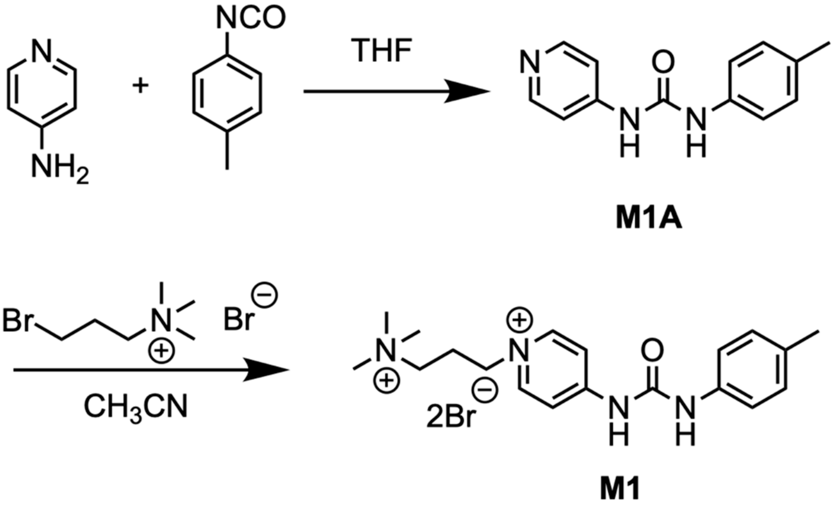

Scheme S1. Synthesis of **M1**

Pyridin-4-amine (0.52 g, 5.50 mmol) and 1-isocyanato-4-methylbenzene (0.66 g, 5.00 mmol) were dissolved in THF (100 mL) and stirred for overnight. Then the solution was poured into diethyl ether to get precipitate. The precipitate was filtered and washed by diethyl ether to afford **M1A**. Compound **M1A** (0.10 g, 0.32 mmol) was dissolved in super dry acetonitrile with a white turbid color. The solution was added to a reflux acetonitrile solution (10 mL) of 3-bromo-N, N, N-trimethylolpropane-1-aminium bromide (0.25 g, 0.96 mmol). The mixture was refluxed for 24 hours, sunk out with the ether, washed several times with acetonitrile, and ether, then dried under vacuum to produce a pure white solid **M1** (0.10 g, 65%). m.p.: 231 °C. ^{1}H NMR (400 MHz, DMSO-d_6, ppm): δ 10.68 (s, 1H, *Ha*), 9.51 (s, 1H, *Hb*), 8.74 (d, J = 7.3 Hz , 2H, *H5*), 7.95 (d, J = 6.9 Hz, 2H, *H6*), 7.40 (d, J = 10.7 Hz, 2H, *H7*), 7.17 (d, J = 8.3 Hz, 2H, *H8*), 4.43 (t, J = 7.2 Hz, 2H, *H4*), 3.05 (s, 9H, *H1*), 2.36 (m, 2H, *H3*), 2.27 (s, 3H, *H9*). ^{13}C NMR (100 MHz, DMSO-d_6, ppm): δ 153.5 (C), 151.5 (CO), 145.2 (CH), 135.8 (C), 133.1 (C), 130.1 (CH), 119.7 (CH), 114.1 (CH), 62.3 (CH), 56.0 (CH), 52.9 (CH), 24.5 (CH), 20.9 (CH). ESI-TOF-MS (*m/z*) [M – 2Br – H]$^+$, calcd. for $[C_{19}H_{27}N_4O]^+$, 327.2179, found 327.2317.

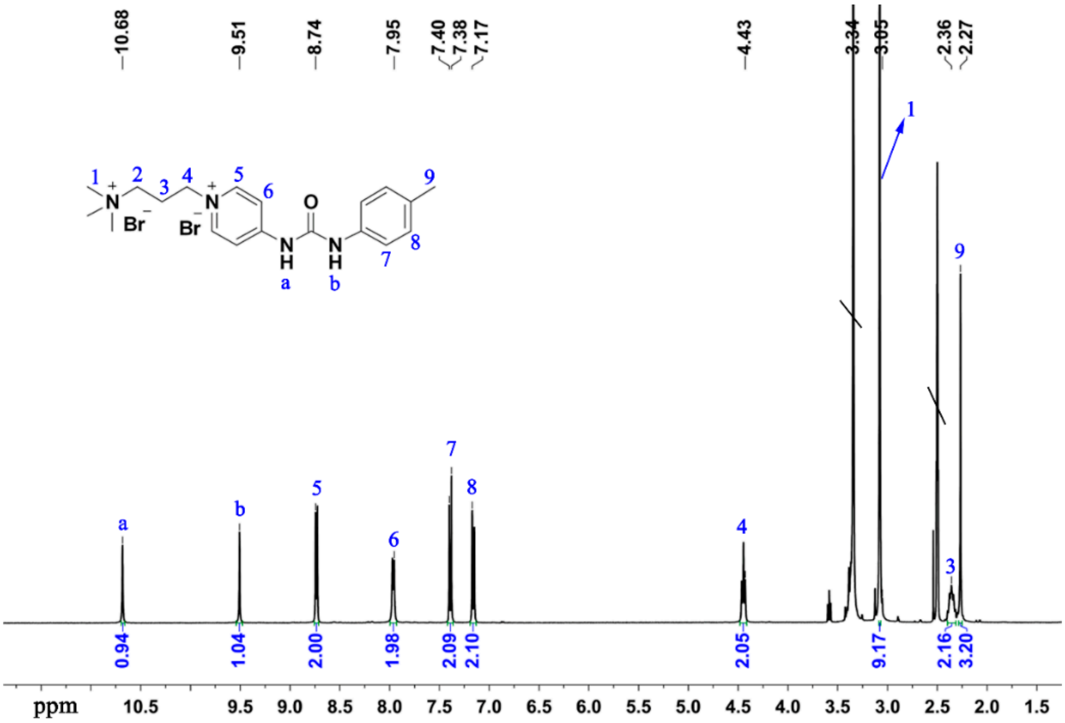

Figure S1. ^{1}H NMR spectrum (400 MHz, DMSO-d_6, 293 K) of compound **M1**

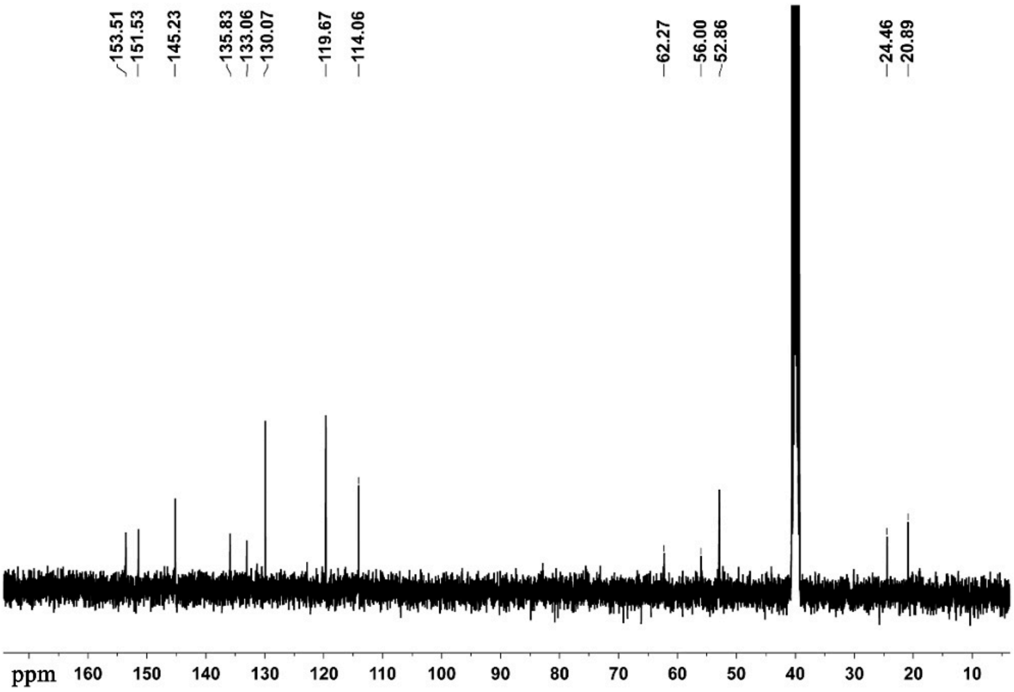

Figure S2. ^{13}C NMR spectrum (100 MHz, DMSO-d_6, 293 K) of compound **M1**.

1.2. Synthesis of M2

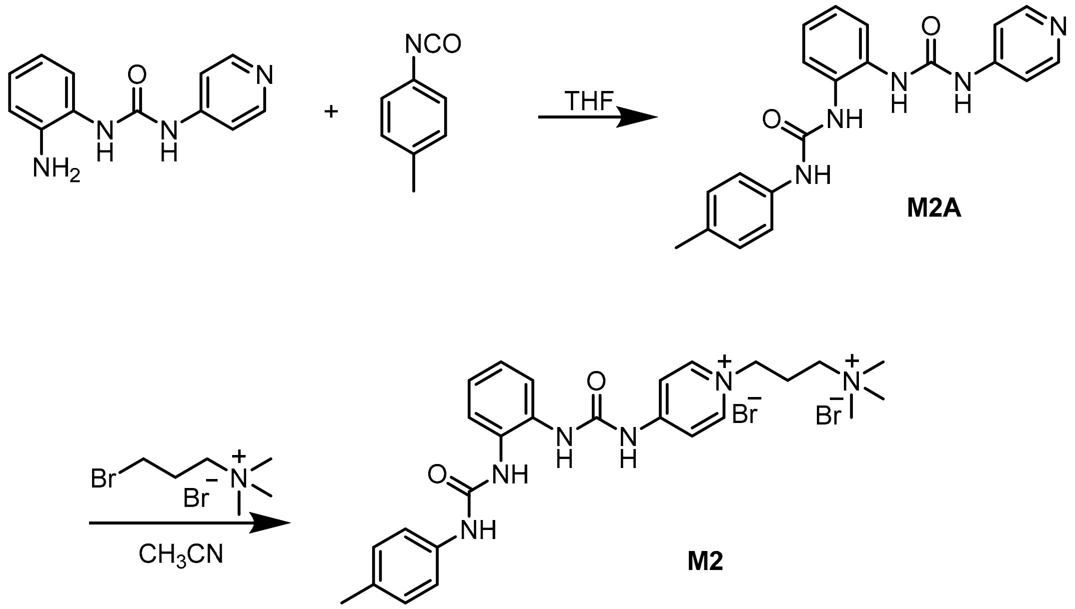

Scheme S2. Synthesis of **M2A, M2**.

1-(2-aminophenyl)-3-(yridine-4-yl) urea (0.10 g, 0.44 mmol) was dissolved in 5 mL THF, 1-isocyanato-4-methylbenzene (0.07 g, 0.53 mmol) was added in drops, refluxed for 6 hours, with white solid precipitated, centrifuged, and washed three times with ether to obtain white solid **M2A** (0.13 g, 80%), m.p.: 260 °C. ^{1}H NMR (400 MHz, DMSO-d_6, ppm): δ 9.52 (s, 1H, *Hd*), 8.94 (s, 1H, *Hc*), 8.35 (d, J = 6.2 Hz, 2H, *H9*), 8.21 (s, 1H, *Hb*), 8.03 (s, 1H, *Ha*), 7.61 (d, J = 7.7 Hz, 1H, *H7*), 7.53 (d, J = 7.4 Hz, 1H, *H4*), 7.44 (d, J = 8.0 Hz, 2H, *H8*), 7.35 (d, J = 8.4 Hz, 2H, *H3*), 7.06 (m, 4H, *H2*, *H5*, *H6*), 2.23 (s, 3H, *H1*). ^{13}C NMR (100 MHz, DMSO-d_6, ppm): δ 153.7 (CO), 153.4 (CO), 150.7 (CH), 147.0 (C), 137.7 (C), 132.5 (C), 131.2 (C), 130.8 (C), 129.7 (CH), 125.2 (C), 125.1 (CH), 124.3 (CH), 124.2 (CH), 118.8 (CH), 112.7 (CH), 20.9 (CH). ESI-TOF-MS (*m/z*) [M + H]$^+$, calcd. for $[C_{20}H_{20}N_5O_2]^+$, 362.1612, found 362.1776.

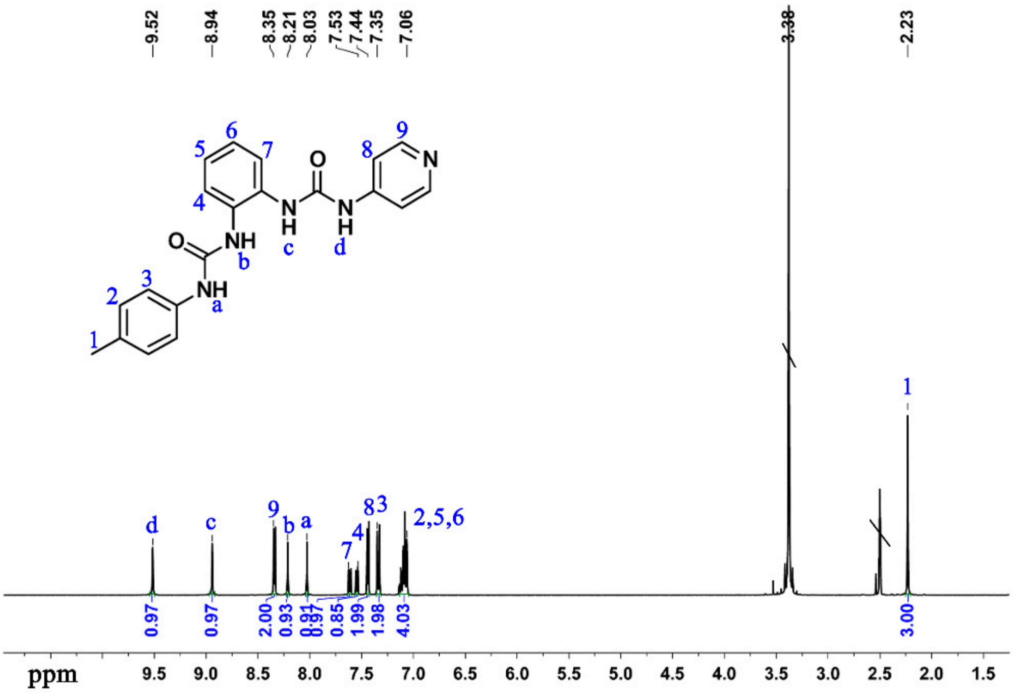

Figure S3. ^{1}H NMR spectrum (400 MHz, DMSO-d_6, 293 K) of compound **M2A**.

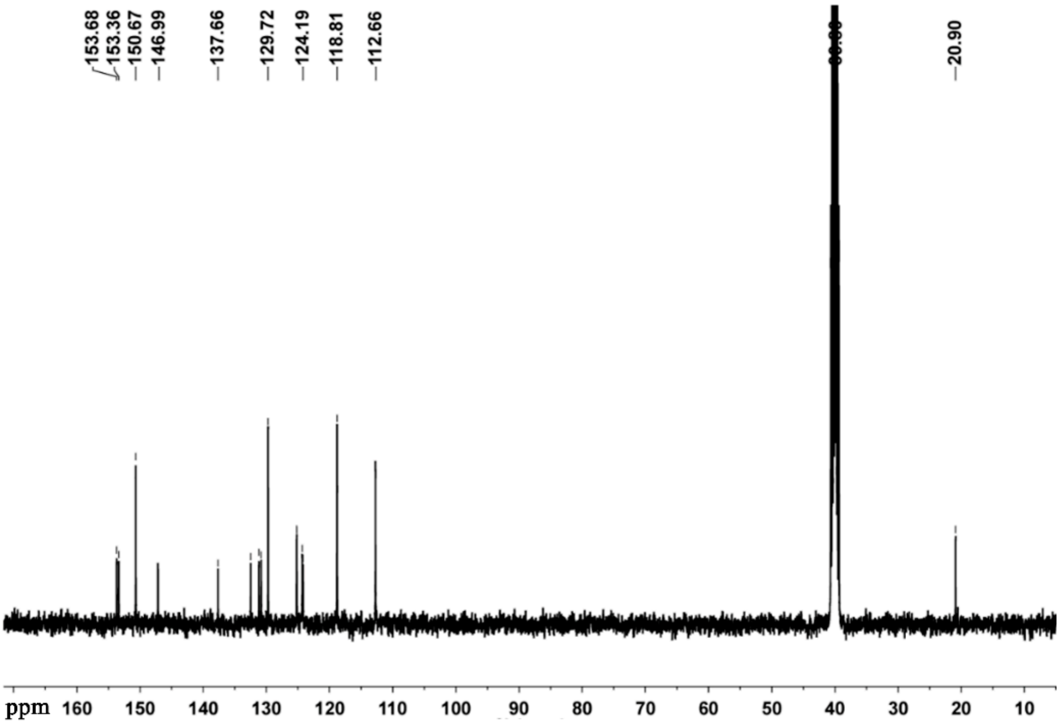

Figure S4. ^{13}C NMR spectrum (100 MHz, DMSO-d_6, 293 K) of compound **M2A**.

Compound **M2A** (0.10g, 0.28 mmol) was dissolved in super dry acetonitrile with a white turbid color. The solution was added to a reflux acetonitrile solution (10mL) of 3-bromo-N, N, N-

trimethylolpropane-1-aminium bromide (1.40 mmol). The mixture was refluxed for 24 hours, sunk out with the ether, washed several times with acetonitrile, ether, then dried under vacuum to produce a pure white solid **M2** (0.11 g, 65%).m.p.: 234 °C. ^{1}H NMR (400 MHz, DMSO-d_6, ppm): δ 10.98 (s, 1H, *Hd*), 9.18 (s, 1H, *Hc*), 8.76 (s, 1H, *Hb*), 8.75 (d, J = 7.0 Hz, 2H, *H9*), 8.33 (s, 1H, *Ha*), 7.96 (d, J = 6.2 Hz, 2H, *H8*), 7.70 (d, J = 8.0 Hz, 1H, *H7*), 7.53 (d, J = 7.8 Hz, 1H, *H4*), 7.36 (d, J = 8.1 Hz, 2H, *H3*), 7.19 (t , J = 7.6 Hz, 1H, *H6*), 7.13 (d, J = 7.7 Hz, 1H, *H5*) , 7.09 (d, J = 8.2 Hz, 2H, *H2*), 4.44 (t, J = 6.9 Hz, 2H, *H10*) , 3.08 (s, 9H, *H13*) , 2.37 (m, 2H, *H12*) , 2.27 (m, 2H, *H11*) , 2.23 (s, 3H, *H1*). ^{13}C NMR (100 MHz, DMSO-d_6, ppm): δ 153.7 (CO), 153.6 (CO), 152.3 (C), 145.1 (C), 137.5 (C), 133.3 (C),131.2 (C), 129.6 (CH), 129.1 (C), 126.3 (CH), 125.7 (CH), 124.0 (CH), 123.5 (CH), 118.7 (CH), 100.0 (CH), 64.5 (CH), 56.0 (CH), 52.7 (CH), 26.0 (CH), 20.8 (CH). ESI-TOF-MS (m/z) $[M - 2Br - H]^+$, calcd. for $[C_{26}H_{33}N_6O_2]^+$, 461.2660, found 461.2840.

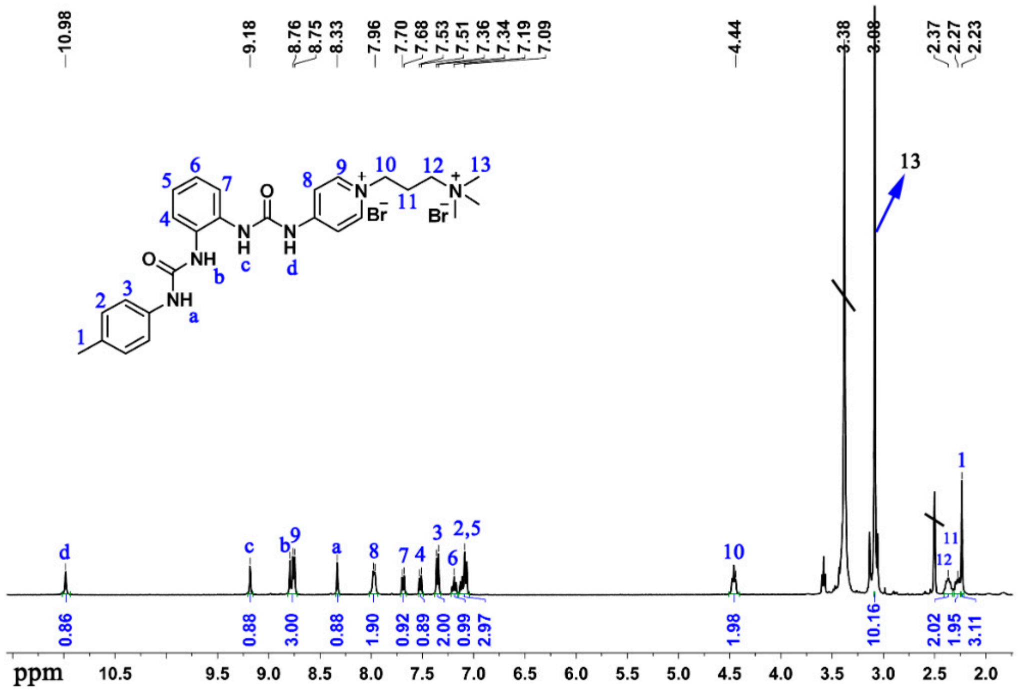

Figure S5. ^{1}H NMR spectrum (400 MHz, DMSO-d_6, 293 K) of compound **M2**.

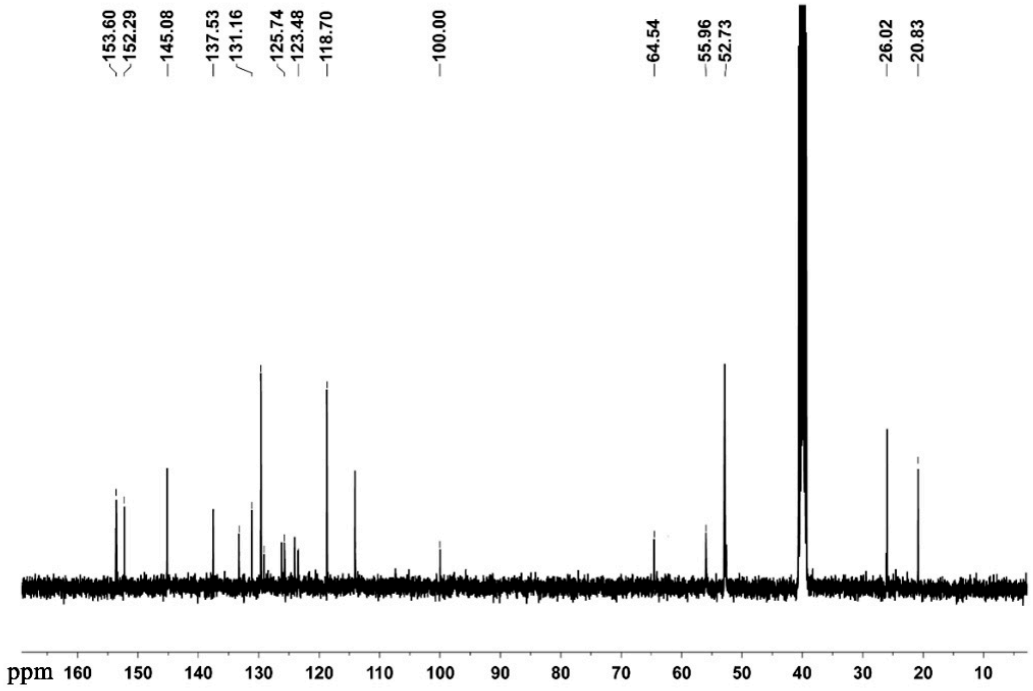

Figure S6. ^{13}C NMR spectrum (100 MHz, DMSO-d_6, 293 K) of compound **M2**.

1.3. Synthesis of M3

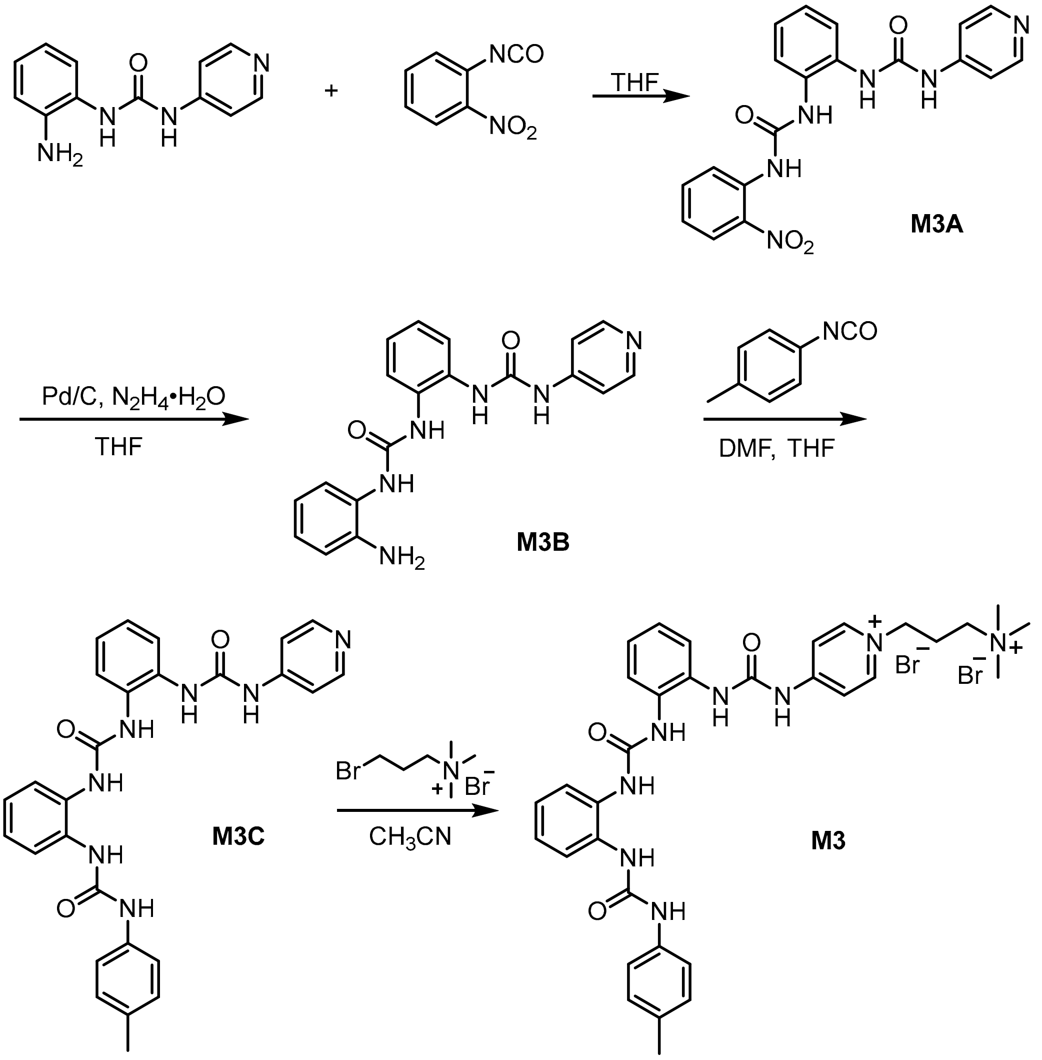

Scheme S3. Synthesis of **M3A, M3B, M3C** and **M3**.

A solution of 1-(2-aminophenyl)-3-(2-nitrophenyl) urea (0.80 g, 3.50 mmol) in 20 mL of THF was added dropwise to a hot solution of bis (4-isocyanatephenyl) methane (0.44 g, 1.75 mmol) in THF (20 mL). After refluxing under intensive stirring for 3 hours, the precipitate was filtered off, washed several times with THF, diethyl ether, then dried in a vacuum to yield analytically pure **M3A** as a white solid (0.98g, 80%), m.p.: 204 °C. ^{1}H NMR (400 MHz, DMSO-d_6, ppm): δ 9.73 (s, 1H, *Hd*), 9.55 (s, 1H, *Hc*), 9.24 (s, 1H, *Hb*), 8.34 (d, J = 6.3 Hz, 2H, *H10*), 8.30 (d, J = 8.2 Hz, 1H, *H8*), 8.22 (s, 1H, *Ha*), 8.10 (d, J = 9.6 Hz, 1H, *H5*), 7.77 (d, J = 7.9 Hz, 1H, *H4*), 7.68 (t, J = 7.9 Hz, 1H, *H7*), 7.42 (m, 3H, *H6, H9*), 7.22 (d, J = 8.2 Hz, 1H, *H1*), 7.17 (d, J = 7.8 Hz, 1H, *H2*), 7.10 (t, J = 6.9 Hz, 1H, *H3*). ^{13}C NMR (100 MHz, DMSO-d_6, ppm): δ 153.4 (CO), 153.0 (CO), 150.7 (C), 147.0 (C), 138.2 (C), 135.4 (C), 135.2 (CH), 132.9 (CH), 129.7 (CH), 126.0 (CH), 125.9 (CH),

124.7 (CH), 123.3 (CH), 122.8 (CH), 112.7 (CH). ESI-TOF-MS (*m/z*) $[M + H]^+$, calcd. for $[C_{19}H_{17}N_6O_4]^+$, 393.1405, found 393.1513.

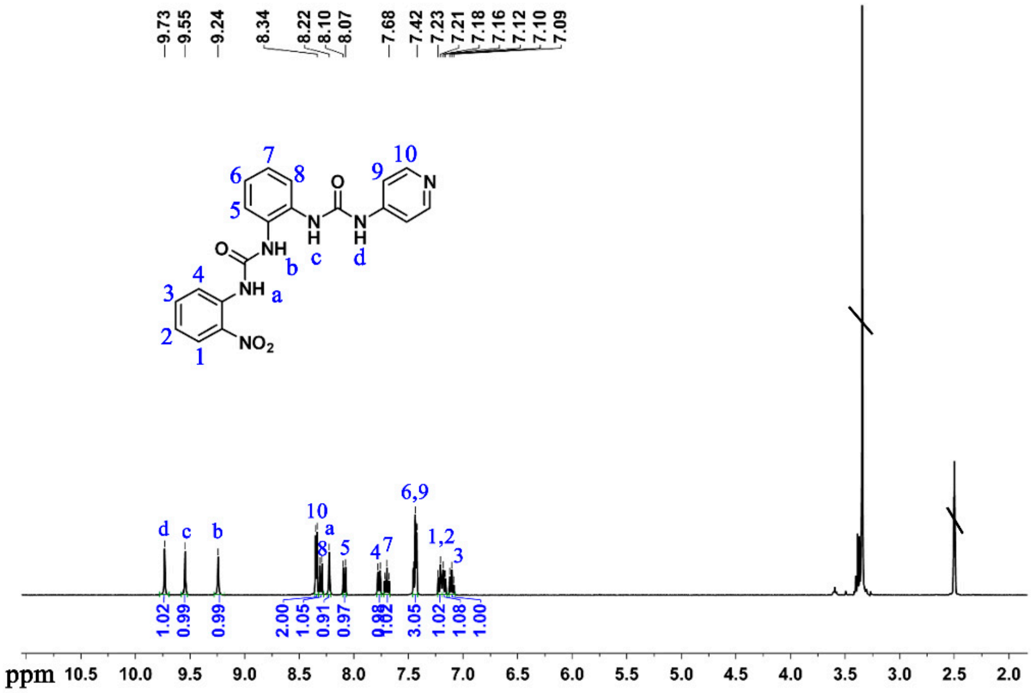

Figure S7. ^{1}H NMR spectrum (400 MHz, DMSO-d_6, 293 K) of compound **M3A.**

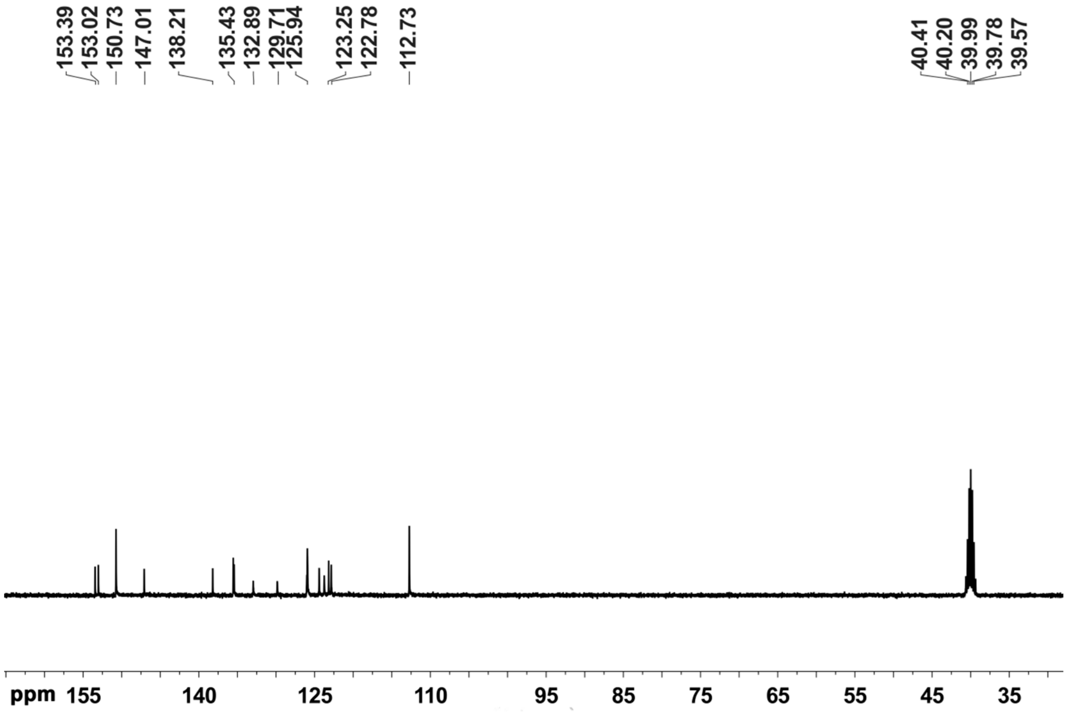

Figure S8. ^{13}C NMR spectrum (100 MHz, DMSO-d_6, 293 K) of compound **M3A.**

To a suspension of **M3A** (0.90 g, 1.40 mmol) and Pd/C 10% (0.09 g, cat.) in THF (50 mL), hydrazine monohydrate (1 mL) was added dropwise. After refluxing under intensive stirring for 5 hours, the solid was filtered off via suction filtration, and then dissolved in DMF (50 mL), filtered through Celite to remove Pd/C. The DMF solution was poured in diethyl ether (200 mL) after which the precipitate thus obtained was filtered off, washed several times with THF, diethyl ether, and dried over a vacuum to give analytically pure **M3B** (0.60 g, 72%) as a white solid, m.p.: > 300 °C. ^{1}H NMR (400 MHz, DMSO-d_6, ppm): δ 9.53 (s, 1H, *He*), 8.34 (d, J = 5.7 Hz, 2H, *H10*), 8.23 (s, 1H, *Hd*), 8.17 (s, 1H, *Hc*), 8.09 (s, 1H, *Hb*), 7.59 (d, J =7 .5 Hz, 1H, *H8*), 7.52 (d, J = 7.4 Hz, 1H, *H5*), 7.44 (d, J = 5.8 Hz, 2H, *H9*), 7.32 (d, J = 7.8 Hz, 1H, *H4*), 7.15- 7.04 (m, 2H, *H6, H7*), 6.84 (t, J = 7.4 Hz, 1H, *H3*), 6.72 (d, J = 7.7 Hz, 1H, *H1*), 6.55 (t, J = 7.4 Hz, 1H, *H2*), 4.81 (s, 2H, *Ha*). ^{13}C NMR (100 MHz, DMSO-d_6, ppm): δ 154.4 (CO), 153.3 (CO), 150.6 (CH), 147.2 (C), 141.7 (C), 132.7 (C), 130.8 (C), 125.3 (C), 125.2 (CH), 125.1 (CH), 124.8 (CH), 124.7 (CH), 124.2 (CH), 124.0 (CH), 117.2 (CH), 116.3 (CH), 112.7 (CH). ESI-TOF-MS (m/z) $[M + H]^+$, calcd. for $[C_{19}H_{19}N_6O_2]^+$, 363.1654, found 363.1758.

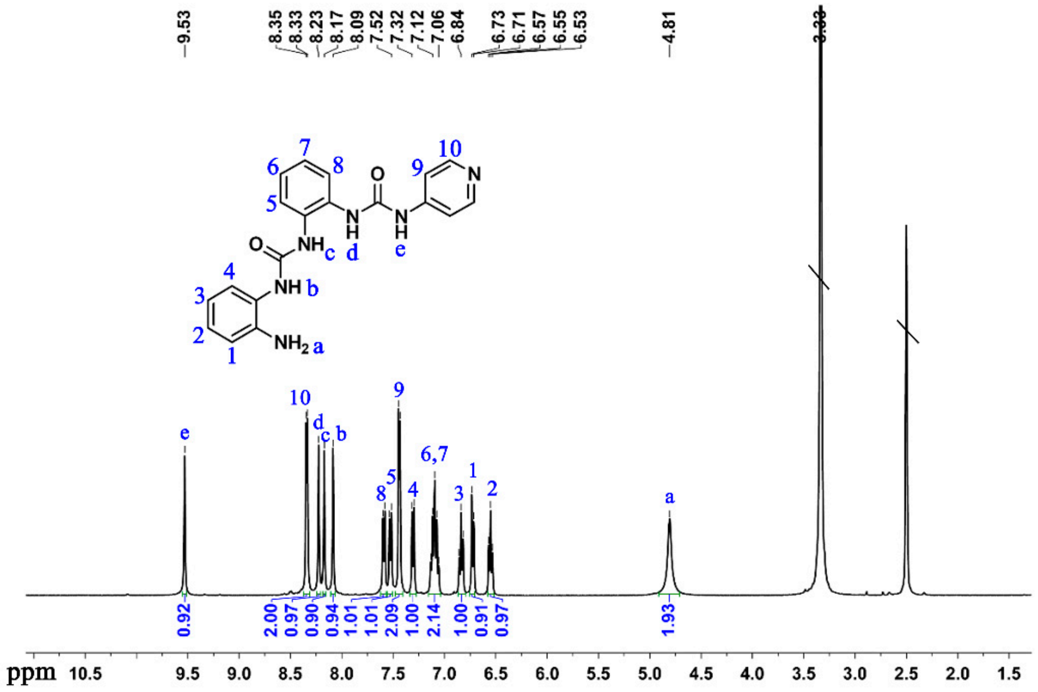

Figure S9. ^{1}H NMR spectrum (400 MHz, DMSO-d_6, 293 K) of compound **M3B**.

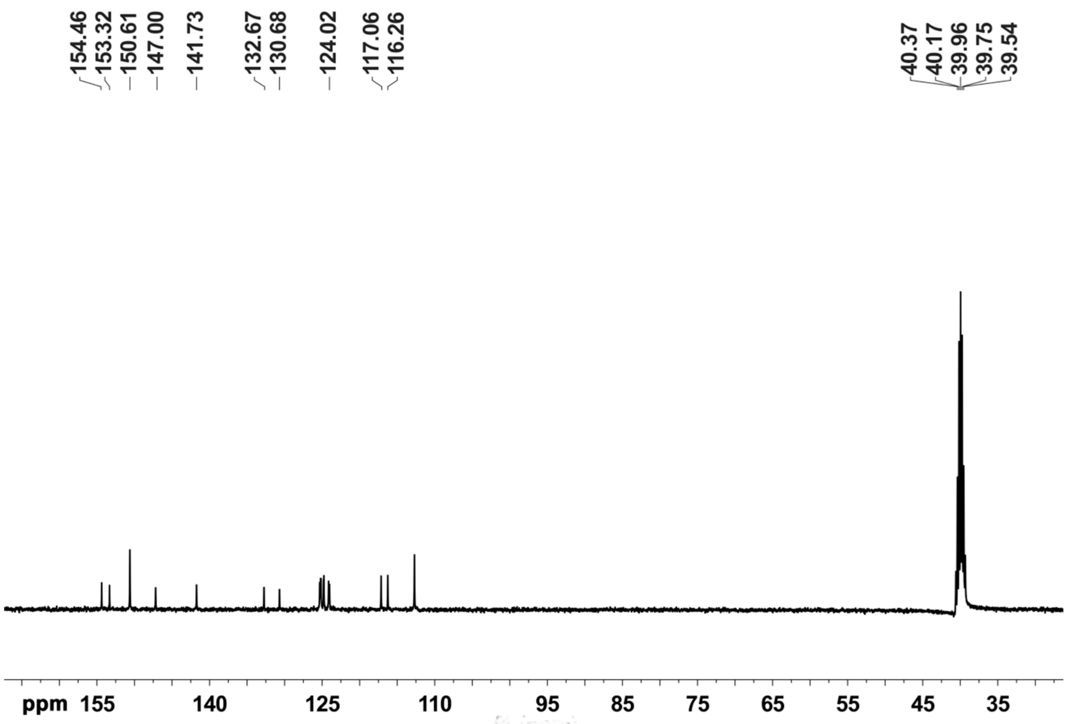

Figure S10. ^{13}C NMR spectrum (100 MHz, DMSO-d_6, 293 K) of compound **M3B.**

A solution of **M3B** (0.30 g, 0.90 mmol) in 2 mL of DMF was added dropwise to a hot solution of p-tolyl isocyanate (0.11 g, 1.00 mmol) in THF (30 mL). After refluxing under intensive stirring for 4 hours, the precipitate was filtered off, washed several times with diethyl ether, and then dried in a vacuum to afford analytically pure **M3C** as a white solid (0.29 g, 71 %), m.p.: > 300 °C. ^{1}H NMR (400 MHz, DMSO-d_6, ppm): δ 9.56 (s, 1H, *Hf*), 8.98 (s, 1H, *He*), 8.47 (s, 1H, *Hd*), 8.43 (s, 1H, *Hc*), 8.32 (d, J = 6.3 Hz, 2H, *H13*), 8.24 (s, 1H, *Hb*), 8.02 (s, 1H, *Ha*), 7.57 (m, 4H, *H8, H9, H10, H11*), 7.41 (d, J = 6.4 Hz, 2H, *H12*), 7.31 (d, J = 8.4 Hz, 2H, *H3*), 7.12 (m, J = 7.8 Hz, 2H, *H2*), 7.06 (m, 4H, *H4, H5, H6, H7*), 2.22 (s, 3H, *H1*). ^{13}C NMR (100 MHz, DMSO-d_6, ppm): δ 154.5 (CO), 153.7 (CO), 153.3 (CO), 150.5 (CH), 147.3 (C), 137.8 (C), 132.2 (C), 132.1 (C), 131.4 (CH), 131.1 (CH), 129.7 (CH), 125.1 (CH), 124.9 (C), 124.8 (C), 124.7 (CH), 124.6 (CH), 124.4 (C), 124.3 (CH), 118.8 (CH), 112.8 (CH), 20.9 (CH). ESI-TOF-MS (*m/z*) $[M + Na]^+$, calcd. for $[C_{27}H_{25}N_7O_3Na]^+$, 518.5906, found 518.5736.

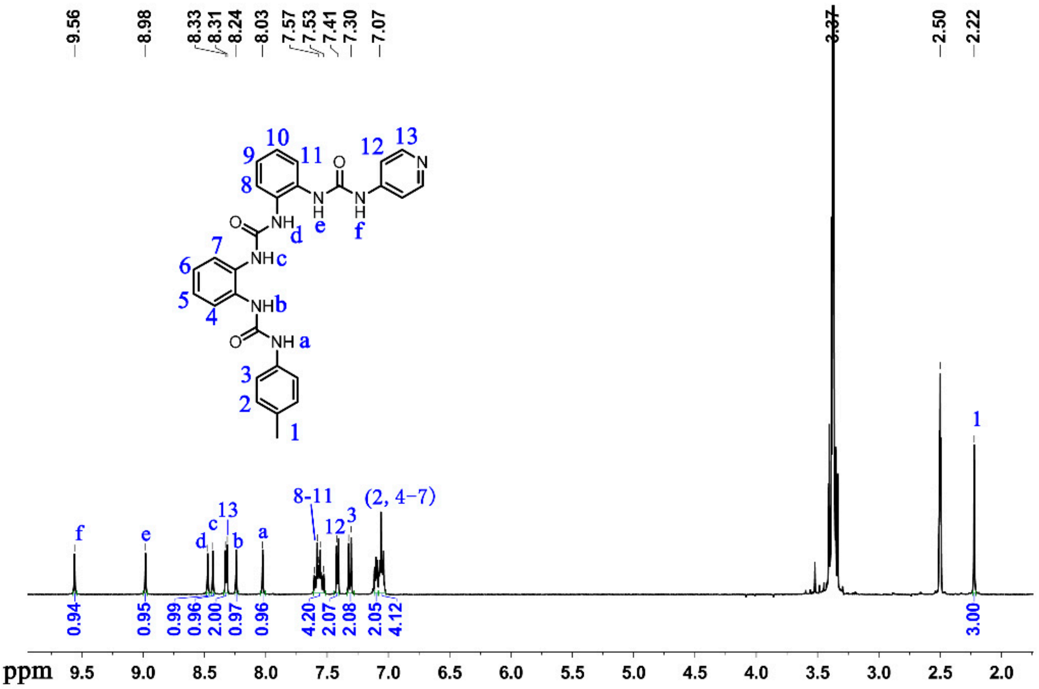

Figure S11. ^{1}H NMR spectrum (400 MHz, DMSO-d_6, 293 K) of compound **M3C.**

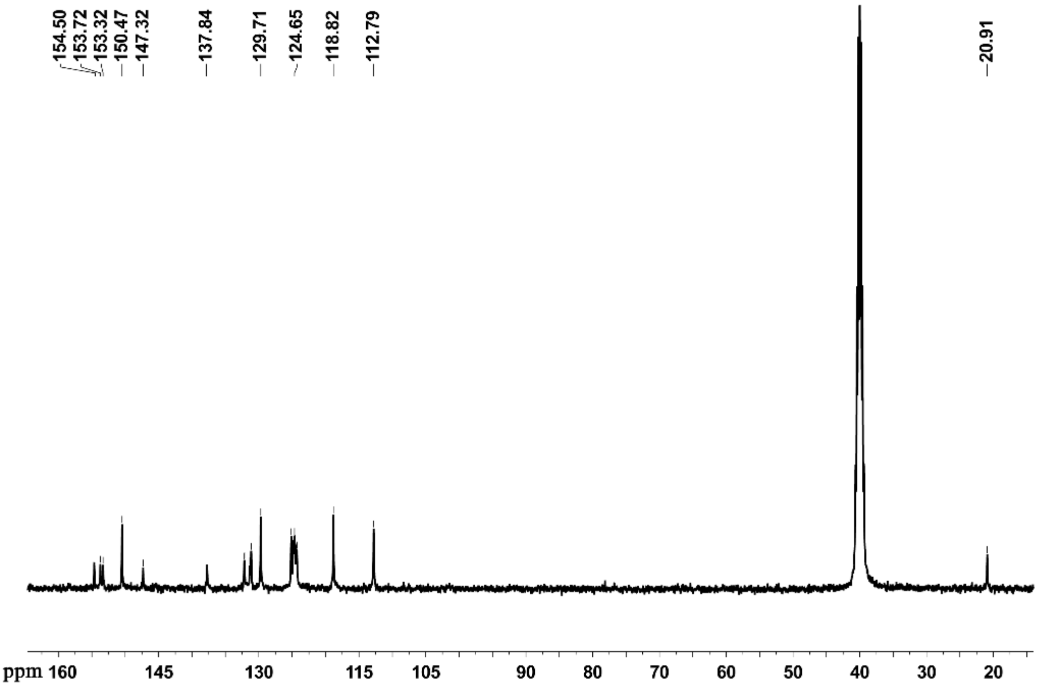

Figure S12. ^{13}C NMR spectrum (100 MHz, DMSO-d_6, 293 K) of compound **M3C.**

Compound **M3C** (0.14 g, 0.32 mmol) was dissolved in 2.5 mL DMF and the solution was added dropwise into a refluxing CH_3CN solution (40 mL) of 3-bromo-N, N, N-trimethylolpropane-1-aminium bromide (0.17 g, 0.64 mmol). The mixture was refluxed for 24 hours, and the precipitate

was filtered off, washed several times with CH_3CN, diethyl ether, and then dried over vacuum to get pure **M3** as a gray solid (0.16 g, 65%), m.p.: > 300 °C. ^{1}H NMR (400 MHz, DMSO-d_6, ppm): δ 11.07 (s, 1H, *Hf*), 9.08 (s, 1H, *He*), 8.83 (s, 1H, *Hd*), 8.68 (d, J = 6.8 Hz, 2H, *H13*), 8.65 (s, 1H, *Hc*), 8,44 (s, 1H, *Hb*), 8.14 (s, 1H, *Ha*), 7.95 (d, J = 8.0 Hz, 2H, *H12*), 7.67 (d, J = 7.4 Hz, 1H, *H11*), 7.59 (m, 3H, *H8, H9, H10*), 7.33 (d, J = 7.8 Hz, 2H, *H3*), 7.18 (m, 2H, *H2*), 7.07 (m, 4H, *H4, H5, H6, H7*), 4.43(t, J = 8.0 Hz, 2H, *H13*), 3.07 (s, 9H, *H17*), 2.33 (m, 4H, *H14, H15*), 2.23 (s, 3H, *H1*). ^{13}C NMR (100 MHz, DMSO-d_6, ppm): δ 143.6 (C), 142.9 (C), 141.9 (C), 136.2 (C), 130.1 (C), 124.3 (C), 119.0 (C), 114.9 (C), 111.3 (C), 62.3 (C), 40.8 (C), 39.7 (C), 36.7 (C). ESI-TOF-MS (*m/z*) $[2M - 2C_6H_{15}Br_2N]^{2+}$, calcd. for $[C_{54}H_{50}N_{14}O_6]^{2+}$, 990.3587, found 990.3876.

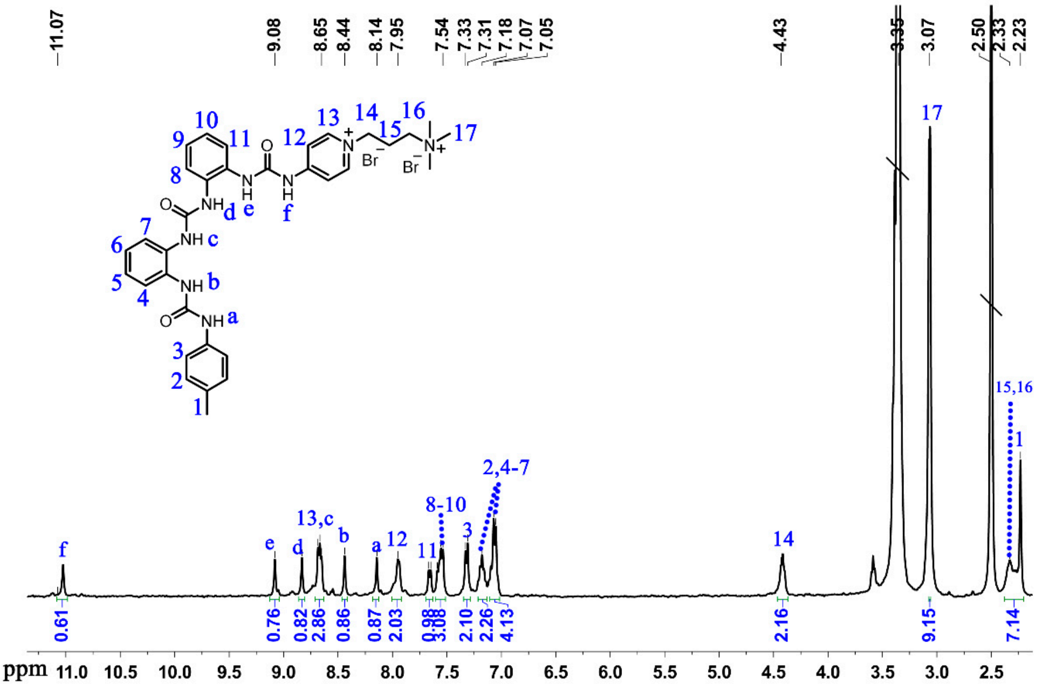

Figure S13. ^{1}H NMR spectrum (400 MHz, DMSO-d_6, 293 K) of compound **M3.**

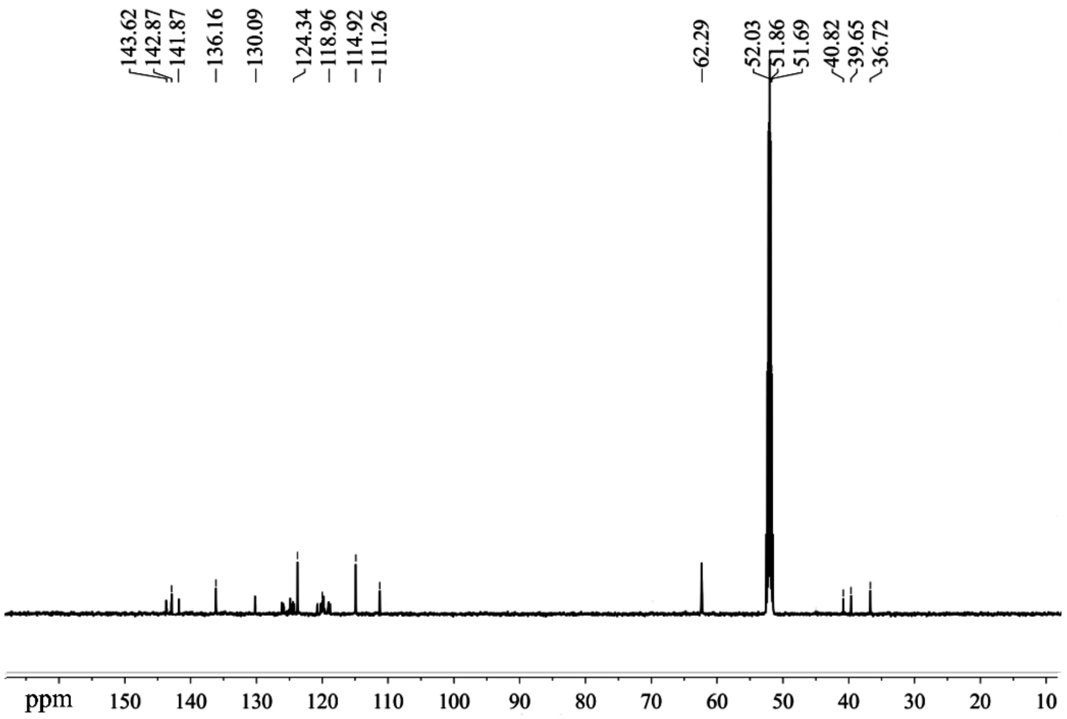

Figure S14. ^{13}C NMR spectrum (100 MHz, DMSO-d_6, 293 K) of compound **M3.**

1.4. Synthesis of D1

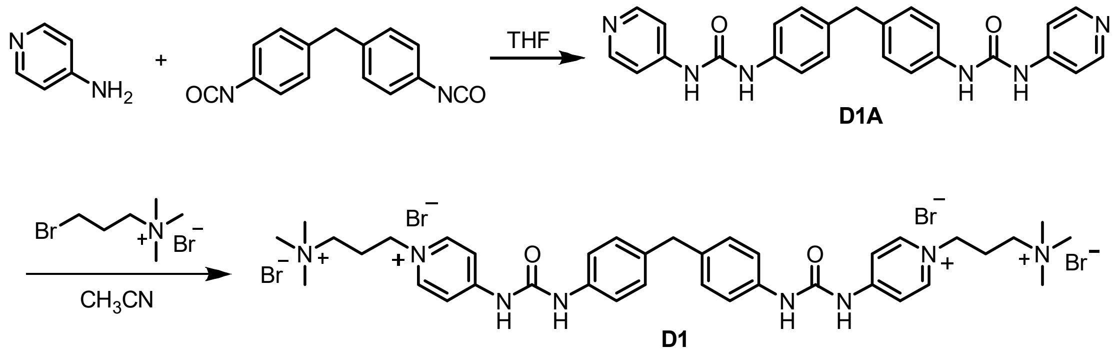

Scheme S4. Synthesis of **D1**.

A solution of 4,4'-methylene bis (phenyl isocyanate) (1.00 g, 4.00 mmol) in 30 mL of THF was added dropwise to a hot solution of pyridine-4-amine (0.76 g, 8.00 mmol) in THF (10 mL). After refluxing under intensive stirring for 2 hours, the precipitate was filtered off, washed several times with THF, and diethyl ether, then dried in a vacuum to yield analytically pure **D1A** as a white solid (1.58 g, yield 90%).

D1A (1.00 g, 1.40 mmol) was dissolved in 5 mL of DMF and the solution was added dropwise into a refluxing CH_3CN solution (50 mL) of 3-bromo-N, N, N-trimethylolpropane-1-aminium bromide (0.93 g, 3.50 mmol). The mixture was refluxed for 8 hours, and the precipitate was filtered

off, washed several times with CH_3CN, diethyl ether, then dried over vacuum to get pure **D1** as a white solid (1.43 g, 83%), m.p.: 221 °C. ^1H NMR (400 MHz, D_2O, ppm): δ 8.49 (d, J = 8.0 Hz, 2H, *H5*), 7.92 (d, J = 8.0 Hz, 2H, *H4*), 7.34 (m, 4H, *H2, H3*), 4.48 (t, J = 8.0 Hz, 2H, *H6*), 3.99 (s, 1H, H1), 3.44 (t, J = 8.0 Hz, 2H, *H8*), 3.17 (s, 9H, *H9*), 2.51 (m, 2H, *H7*). ^{13}C NMR (100 MHz, D_2O, ppm): δ 153.0 (CO), 151.4 (C), 143.9 (CH), 137.7 (C), 134.9 (C), 129.3 (CH), 120.2 (CH), 114.3 (CH), 62.5 (CH), 55.8 (CH), 53.3 (CH), 24.5 (CH). ESI-TOF-MS (*m/z*) $[M - 3Br]^{3+}$, calcd. for $[C_{37}H_{52}BrN_8O_2]^{3+}$, 719.3391, found 719.3592.

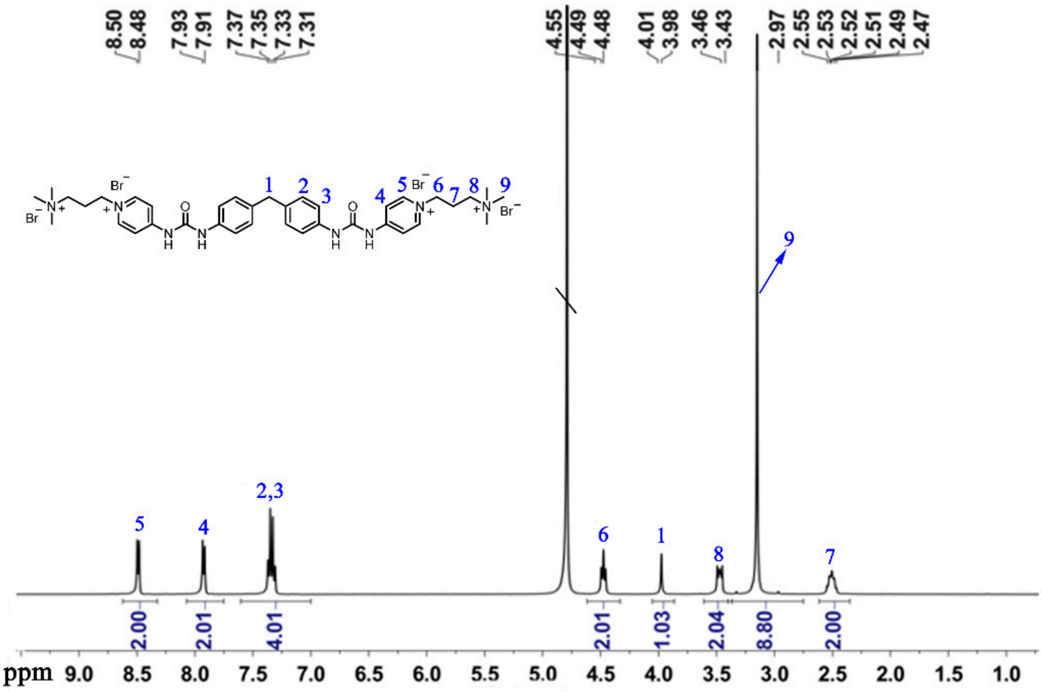

Figure S15. ^1H NMR spectrum (400 MHz, D_2O, 293 K) of compound **D1.**

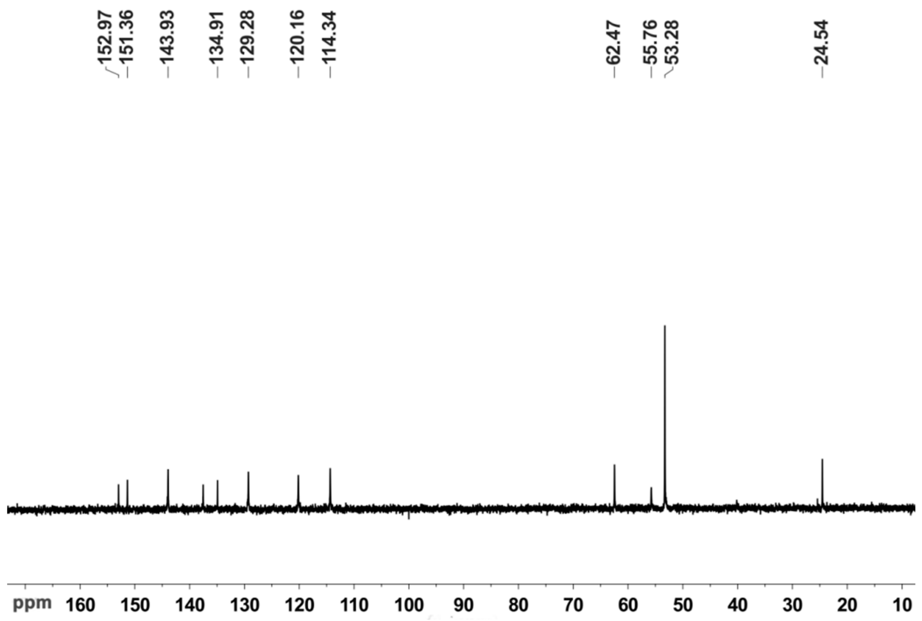

Figure S16. ^{13}C NMR spectrum (100 MHz, D_2O, 293 K) of compound **D1**.

1.5. Synthesis of D2

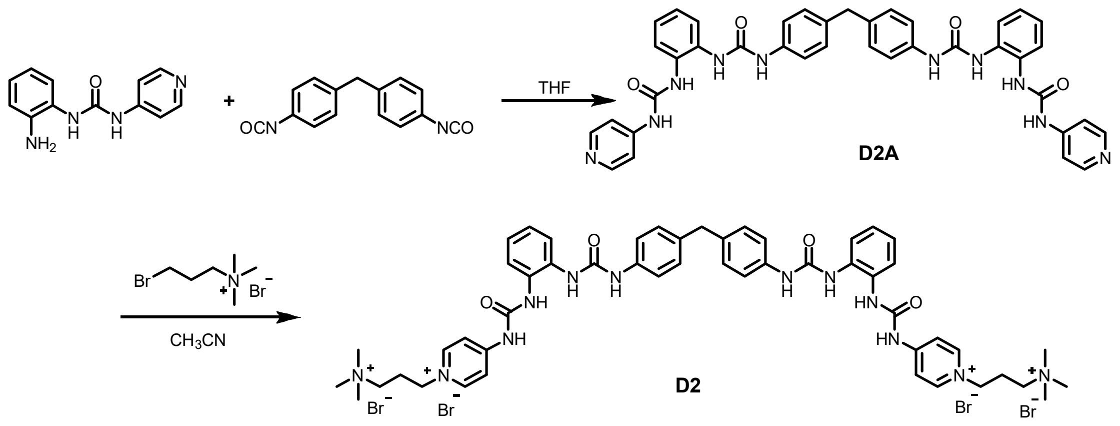

Scheme S5. Synthesis of **D2A, D2**.

A solution of 4,4'-methylene bis (phenyl isocyanate) (1.00 g, 4.00 mmol) in 30 mL of THF was added dropwise to a hot solution of 1-(2-aminophenyl)-3-(pyridin-4-yl) urea (1.82 g, 8.0 mmol) in THF (40 mL). After refluxing under intensive stirring for 2 hours, the precipitate was filtered off, washed several times with THF, diethyl ether, then dried in vacuum to yield analytically pure **D2A** as a white solid (2.54 g, 85%), m.p.: > 300 °C. ^{1}H NMR (400 MHz, DMSO-d_6, ppm): δ 9.51 (s, 1H, *Hd*), 9.00 (s, 1H, *Hc*), 8.34 (d, J = 6.4 Hz, 2H, *H9*), 8.20 (s,1H, *Hb*), 8.04 (s,1H, *Ha*), 7.63 (d, J = 7.8Hz, 1H, *H7*), 7.53 (d, J = 7.7 Hz, 1H, *H4*), 7.43 (d, J = 6.3 Hz, 2H, *H8*), 7.35 (d, J = 8.5 Hz, 2H, *H3*), 7.14 - 7.05 (m, 4H, *H2, H5, H6*), 3.80 (s, 1H, *H1*). ^{13}C NMR (100 MHz, DMSO-d_6, ppm): δ

153.6 (CO), 153.4 (CO), 150.7 (CH), 147.1 (C), 138.1 (C), 135.5 (C), 132.5 (C), 130.6 (C), 129.5 (CH), 125.3 (CH), 125.2 (CH), 124.3 (CH), 124.0 (CH), 118.9 (CH), 112.7 (CH). ESI-TOF-MS (*m/z*) $[M + H]^+$, calcd. for $[C_{39}H_{35}N_{10}O_4]^+$, 707.2857, found 707.2907.

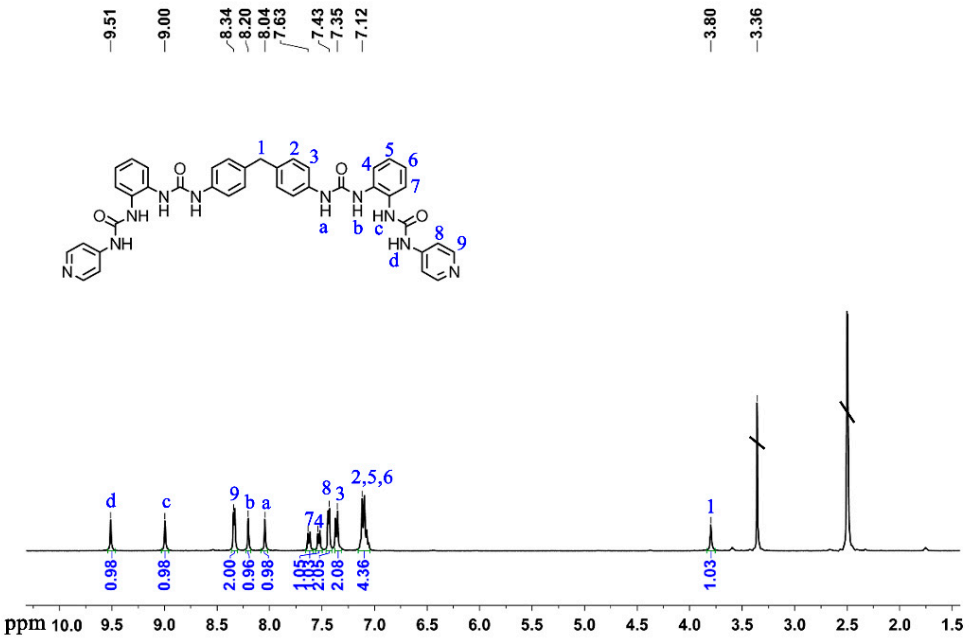

Figure S17. ^{1}H NMR spectrum (400 MHz, DMSO-d_6, 293 K) of compound **D2A**.

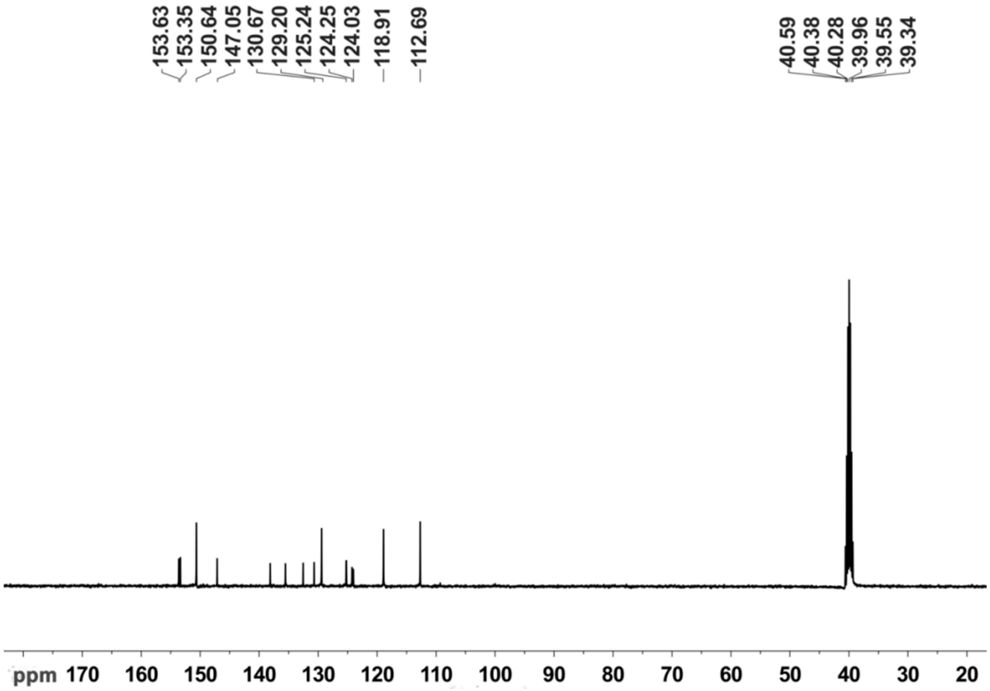

Figure S18. ^{13}C NMR spectrum (100 MHz, DMSO-d_6, 293 K) of compound **D2A**.

Compound **D2A** (1.00 g, 1.30 mmol) was dissolved in 8.0 mL DMF and the solution was added dropwise into a refluxing CH_3CN solution (50 mL) of 3-bromo-N, N, N-trimethylolpropane-1-aminium bromide (0.86 g, 3.30 mmol). The mixture was refluxed for 12 hours, and the precipitate was filtered off, washed several times with CH_3CN, ether, then dried over vacuum to get pure **D2** as a gray solid (1.27 g, 75%). m.p.:209 °C. ^{1}H NMR (400 MHz, D_2O, ppm): δ 8.41 (d, J = 8.31 Hz, 2H, *H9*), 7.84 (d, J = 7.74 Hz, 2H, *H8*), 7.53 (t, J = 7.44 Hz, 1H, *H6*), 7.34 (t, J = 7.38 Hz, 1H, *H5*), 7.22 (m, 2H, *H4, H7*), 7.17 (m, J = 7.09 Hz, 4H, *H2, H3*), 4.37 (t, J = 8.0 Hz, 2H, *H10*), 3.79 (s, 1H, *H1*), 3.41 (t, J = 8.0 Hz, 2H, *H12*), 3.09 (s, 9H, *H13*), 2.41 (m, 2H, *H11*). ^{13}C NMR (100 MHz, DMSO-d_6, ppm): δ 153.6 (CO), 152.4 (C), 145.3 (CH), 138.1 (C), 135.7 (C), 133.5 (C), 129.5 (CH), 129.0 (CH), 126.5 (CH), 125.9 (CH), 124.1 (CH), 123.4 (CH), 118.9 (CH), 114.0 (CH), 62.2 (CH), 55.9 (CH), 52.9 (CH), 26.0 (CH), 24.6 (CH). ESI-TOF-MS (m/z) [M – Br]$^+$, calcd. for $[C_{51}H_{64}Br_3N_{12}O_4]^+$, 1149.2690, found 1149.2796.

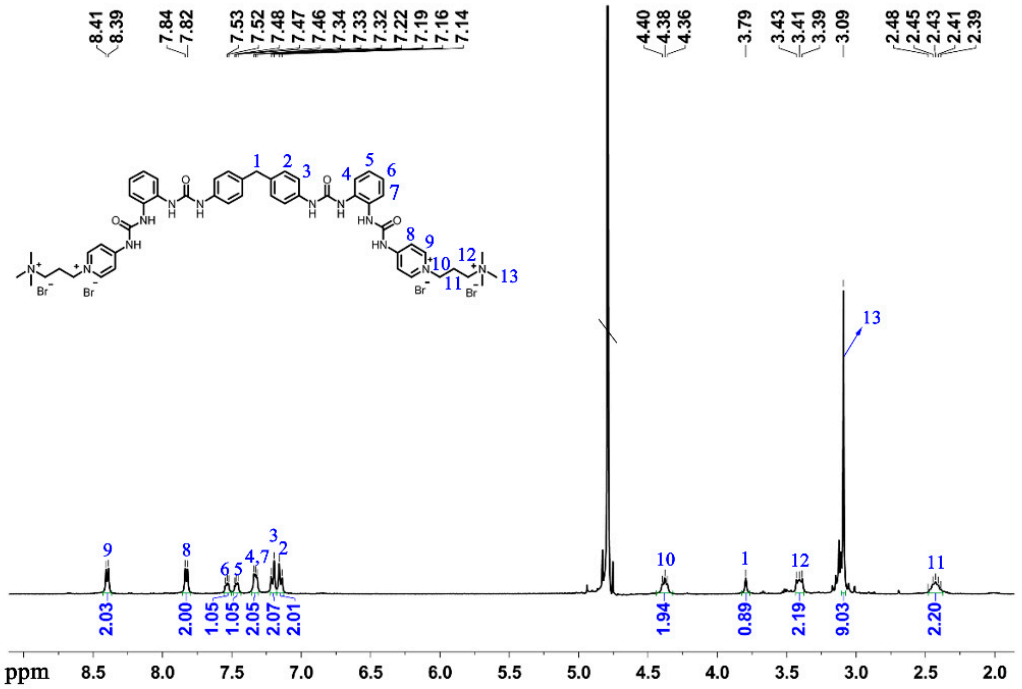

Figure S19. ^{1}H NMR spectrum (400 MHz, D_2O, 293 K) of compound **D2.**

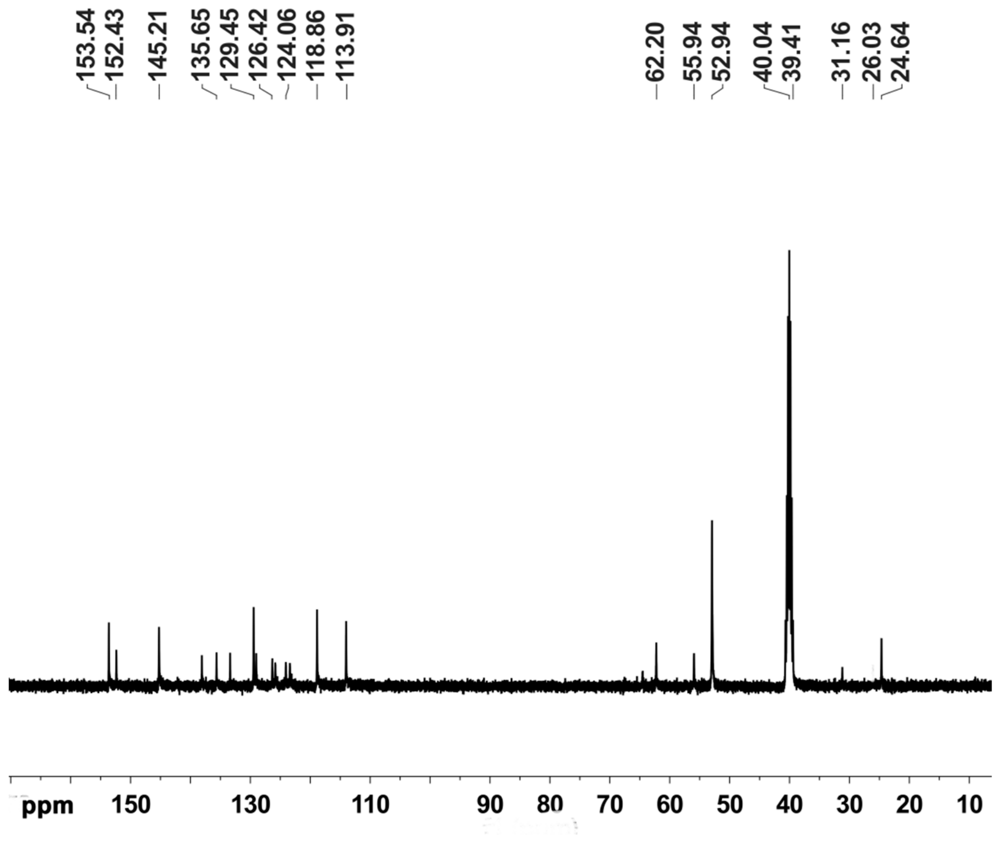

Figure S20. ^{13}C NMR spectrum (100 MHz, DMSO-d_6, 293 K) of compound **D2**.

1.6. Syntheses of D3

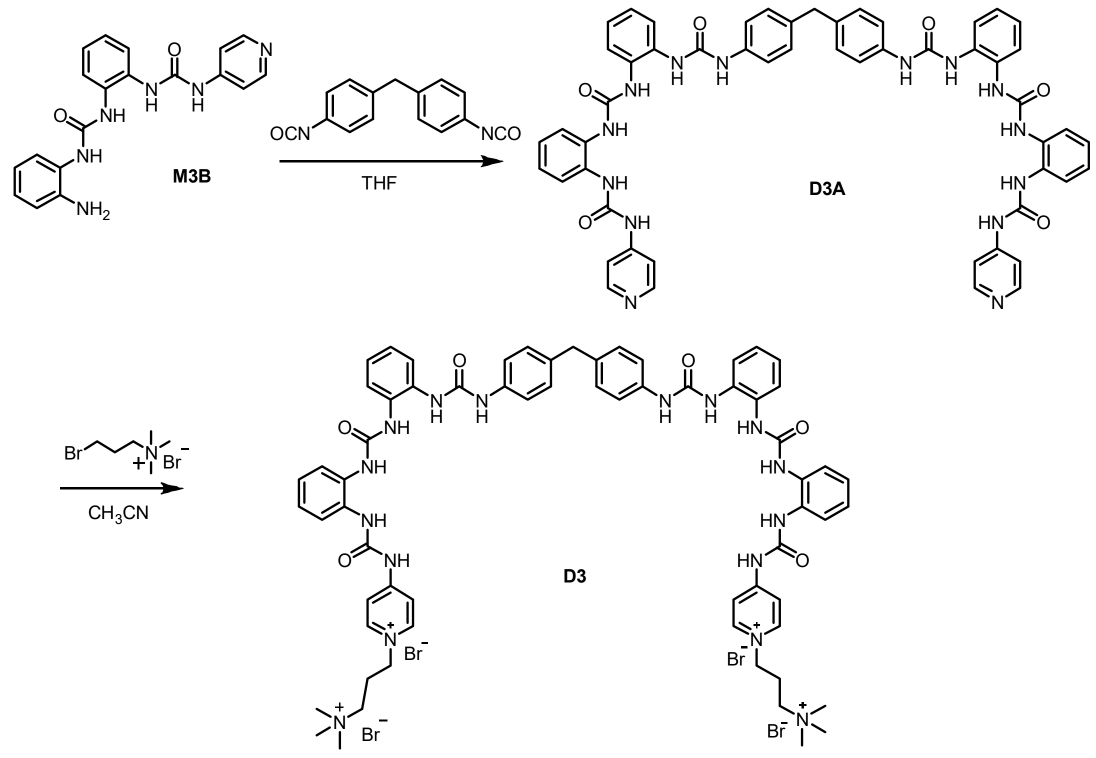

Scheme S6. Synthesis of **D3A**, and **D3**.

A solution of **M3B** (0.60 g, 0.80 mmol) in 2 mL of DMF was added dropwise to a hot solution of *o*-nitrophenyl isocyanate (0.26 g, 1.60 mmol) in THF (20 mL). After refluxing under intensive stirring for 2.5 hours, the precipitate was filtered off, washed several times with THF, diethyl ether, then dried in a vacuum to obtain analytically pure **D3A** as a white solid (0.6 g, 73 %), m.p.: > 300 °C. ^{1}H NMR (400 MHz, DMSO-d_6, ppm): δ 9.55 (s, 1H, *Hf*), 9.02 (s, 1H, *He*), 8.47 (s, 1H, *Hd*), 8.43 (s, 1H, *Hc*), 8.32 (d, J = 4.4 Hz, 2H, *H13*), 8.23 (s, 1H, *Hb*), 8.03 (s, 1H, *Ha*), 7.56 (m, 4H, *H8, H9, H10, H11*), 7.42 (d, J = 4.5 Hz, 2H, *H12*), 7.33 (d, J =7.4 Hz, 2H, *H3*), 7.10 (m, 6H, *H2, H4, H5, H6, H7*), 3.78 (s, 1H, *H1*). ^{13}C NMR (100 MHz, DMSO-d_6, ppm): δ 154.6 (CO), 153.6 (CO), 153.3 (CO), 150.6 (CH), 147.1 (C), 138.1 (C), 135.5 (C), 132.3 (C), 132.1 (C), 131.2 (C), 131.1 (CH), 129.4 (C), 125.1 (CH), 125.0 (CH), 124.9 (CH), 124.8 (CH), 124.5 (CH), 124.3 (CH), 124.2 (CH), 118.9 (CH), 118.8 (CH), 112.7 (CH). ESI-TOF-MS (*m/z*) [M + H]$^+$, calcd. for $[C_{53}H_{47}N_{14}O_6]^+$, 975.3879, found 975.3909.

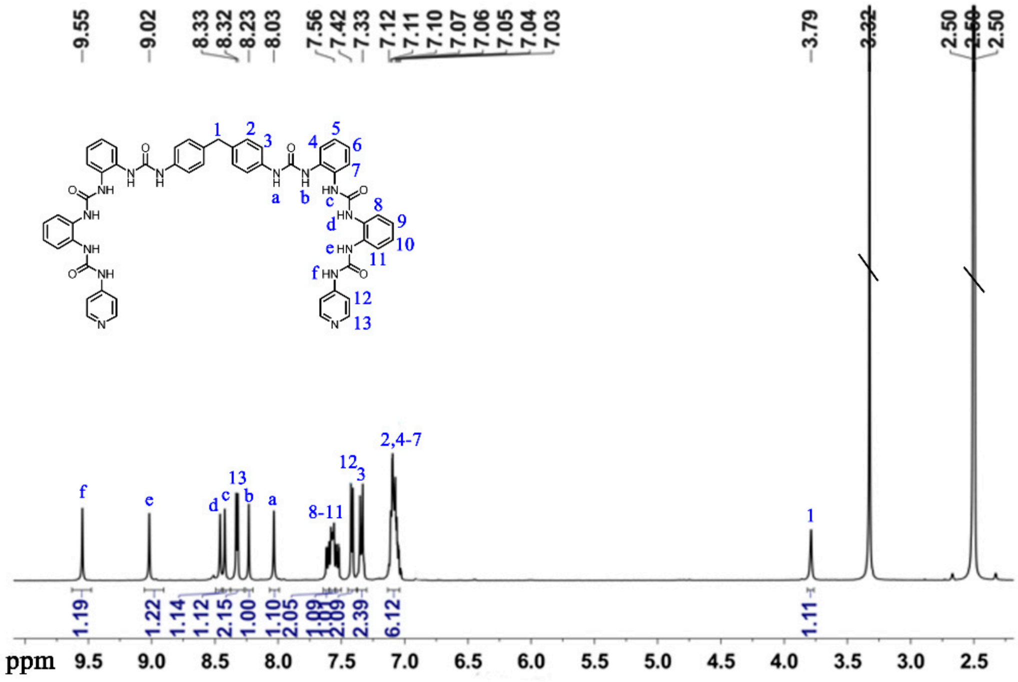

Figure S21. ^{1}H NMR spectrum (400 MHz, DMSO-d_6, 293 K) of compound **D3A.**

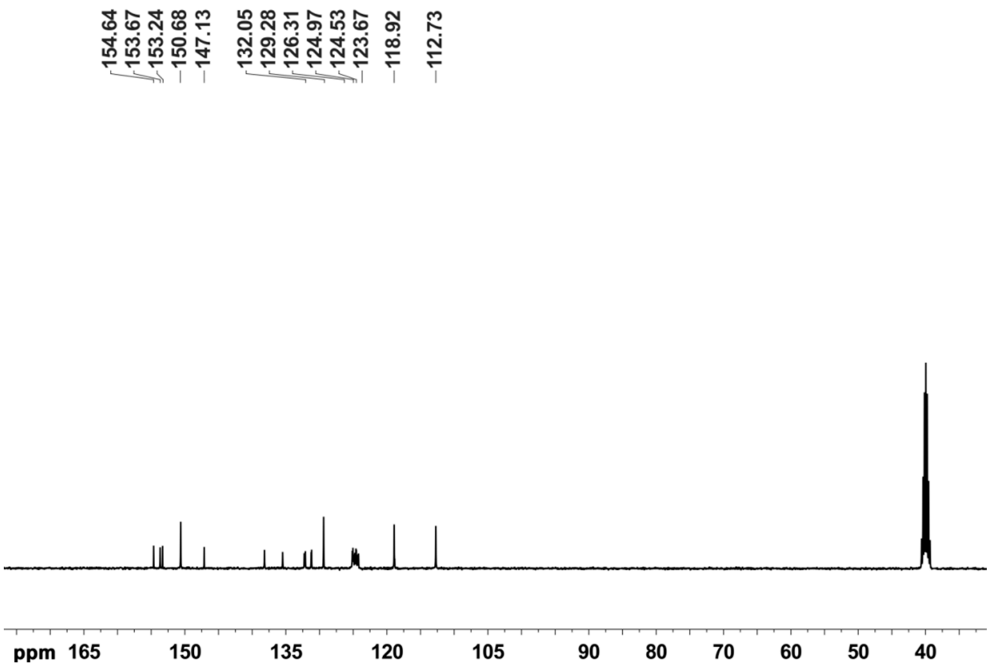

Figure S22. ^{13}C NMR spectrum (100 MHz, DMSO-d_6, 293 K) of compound **D3A.**

Compound **D3A** (0.60 g, 0.58 mmol) was dissolved in 2.5 mL DMF and the solution was added dropwise into a refluxing CH_3CN solution (40 mL) of 3-bromo-N, N, N-trimethylolpropane-1-aminium bromide (0.76 g, 2.90 mmol). The mixture was refluxed for 8 hours, and the precipitate was filtered off, washed several times with CH_3CN, diethyl ether, then dried over vacuum to get pure **D3** as a gray solid (0.54 g, 60%), m.p.: 205 °C. ^1H NMR (400 MHz, DMSO-d_6, ppm): δ 11.05 (s, 1H, *Hf*), 9.16 (s, 1H, *He*), 8.84 (s, 1H, *Hd*), 8.69 (d, J = 6.6 Hz, 2H, *H13*), 8.64 (s, 1H, *Hc*), 8.46 (s, 1H, *Hb*), 8.02 (s, 1H, *Ha*), 7.95 (d, J = 8.0 Hz, 2H, *H12*), 7.66 (t, J = 7.3 Hz, 1H, *H6*), 7.52 (m, 3H, *H8, H9, H10*), 7.35 (d, J = 8.1 Hz, 2H, *H3*), 7.19 (d, J = 8.2 Hz, 2H, *H2*), 7.05 (m, 4H*, H4, H5, H6, H7*), 4.41 (t, J = 6.8 Hz, 2H, *H14*), 3.80 (s, 1H, *H1*), 3.06 (s, 11H, *H15, H17*), 2.38 - 2.25 (m, 2H, *H16*). ^{13}C NMR (100 MHz, DMSO-d_6, ppm): δ 154.7 (CO), 153.6 (CH), 152.3 (CO), 145.2 (CH), 138.2 (CO), 135.5 (C), 132.8 (C), 132.3 (C), 130.1 (C), 129.5 (CH), 125.9 (C), 125.4 (C), 125.0 (CH), 124.9 (CH), 124.8 (CH), 123.8 (CH), 123.3 (CH), 118.8 (CH), 114.0 (CH), 62.3 (CH), 56.0 (CH), 53.0 (CH), 24.65 (CH). ESI-TOF-MS (*m/z*) $[2M - 2Br + CH_3CN + H_2O]^{2+}$, calcd. for $[C_{131}H_{157}Br_6N_{33}O_{13}]^{2+}$, 1446.6826, found 1446.6682.

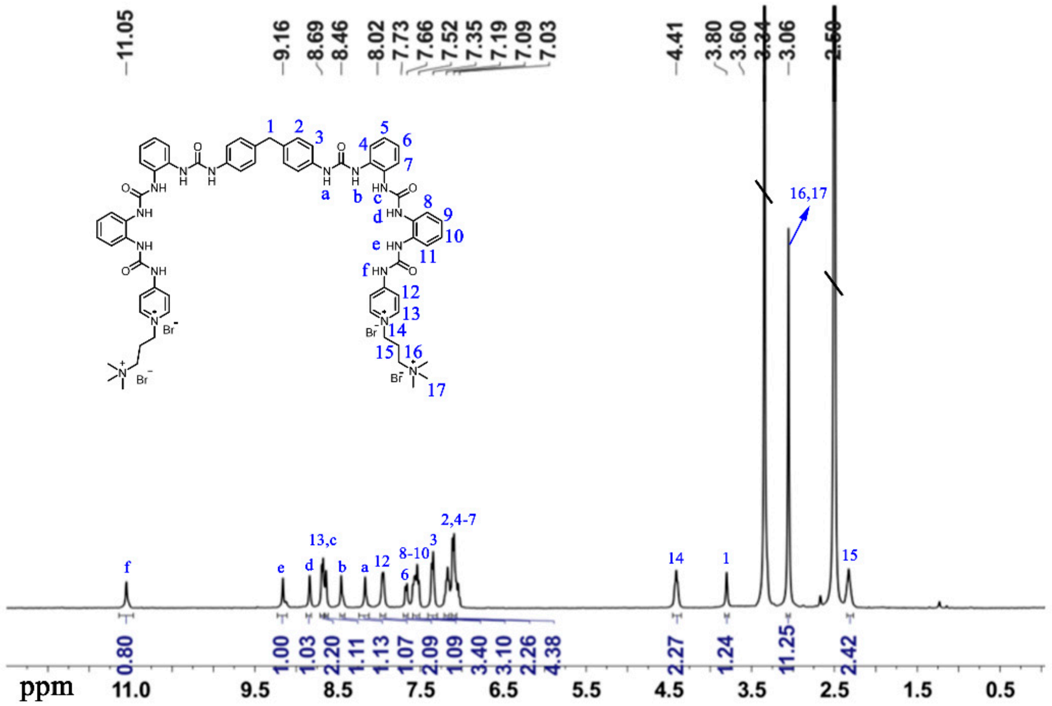

Figure S23. ^{1}H NMR spectrum (400 MHz, DMSO-d_6, 293 K) of compound **D3.**

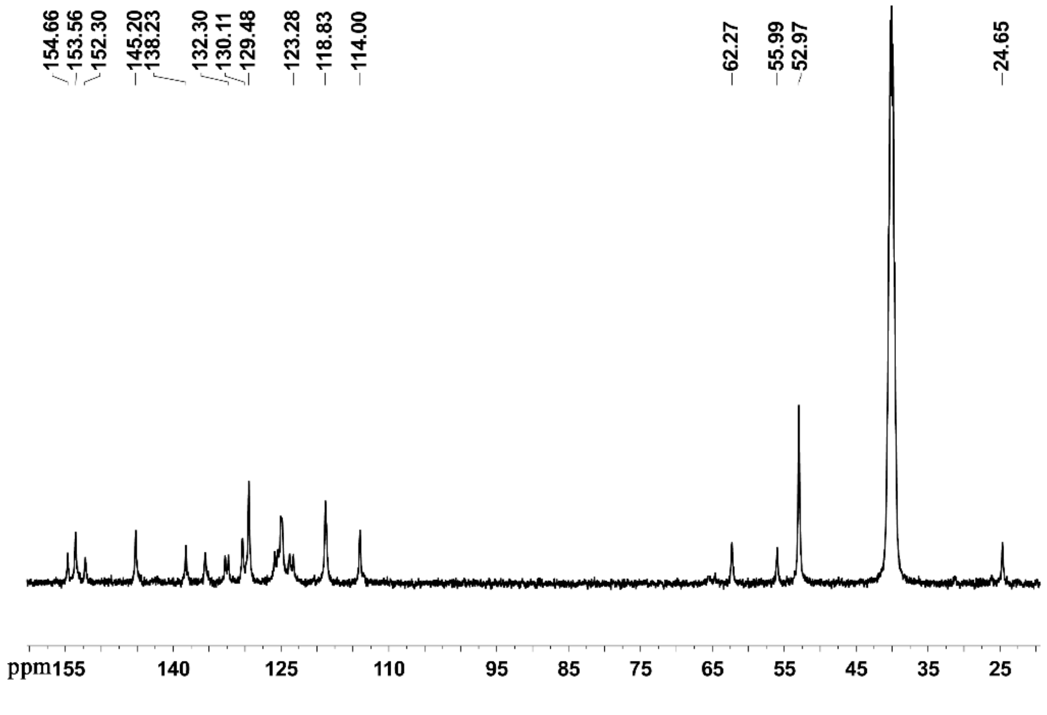

Figure S24. ^{13}C NMR spectrum (100 MHz, DMSO-d_6, 293 K) of compound **D3**.

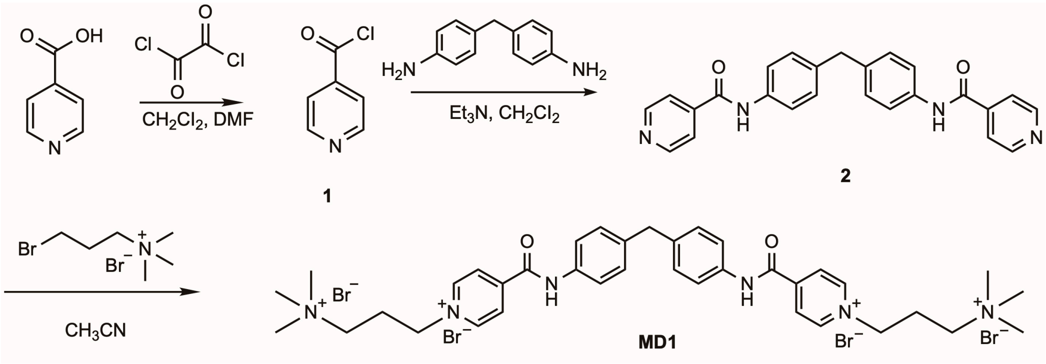

Scheme S7. Synthesis of **MD1**.

Oxalyl dichloride (1.47 g, 1.33 mmol) was diluted with CH_2Cl_2 (5 mL). Isonicotinic acid (0.61 g, 0.50 mmol) was added into the CH_2Cl_2 solution. Then 30 mL DMF was further added into the solution. The mixture was refluxed for 6 h. The solution was dried over vacuum to get compound **1**. Subsequently, a mixture of 4,4'-methylenedianiline (0.29 g, 1.44 mmol) and triethylamine (0.36 g, 3.6 mmol) was added into the CH_2Cl_2 solution of compound **1** to generate yellow precipitate. The precipitate was filtered and washed with water for three times to afford compound **2**. **2** (0.023 g, 0.056 mmol) was dissolved in 5 mL of DMF and the solution was added dropwise into a refluxing CH_3CN solution (50 mL) of 3-bromo-N, N, N-trimethylolpropane-1-aminium bromide (0.12 g, 0.45 mmol). The mixture was refluxed for 8 hours, and the precipitate was filtered off, washed several times with CH_3CN, diethyl ether, then dried over vacuum to get pure **MD1** as a white solid (0.04 g, 74%). 1H NMR (400 MHz, DMSO-d_6, ppm): δ 10.98 (s, 1H, *Ha*), 9.36 (d, J = 6.7 Hz, 2H, *H5*), 8.64 (d, J = 6.8 Hz, 2H, *H4*), 7.75 (d, J = 8.5 Hz, 2H, *H3*), 7.30 (d, J = 8.5 Hz, 2H, *H2*), 4.77 (t, J = 7.3 Hz, 2H, *H6*), 3.96 (s, 1H, *H1*), 3.44 (d, J = 11.6 Hz, *H8*), 3.10 (s, 9H, *H9*). ^{13}C NMR (100 MHz, DMSO-d_6, ppm): δ 160.69 (CO), 148.82 (C), 145.90 (CH), 138.00 (C), 136.00 (CH), 128.88 (CH), 126.19 (C), 120.79 (CH), 61.65 (CH), 52.62 (CH), 52.41 (CH), 24.20 (CH).

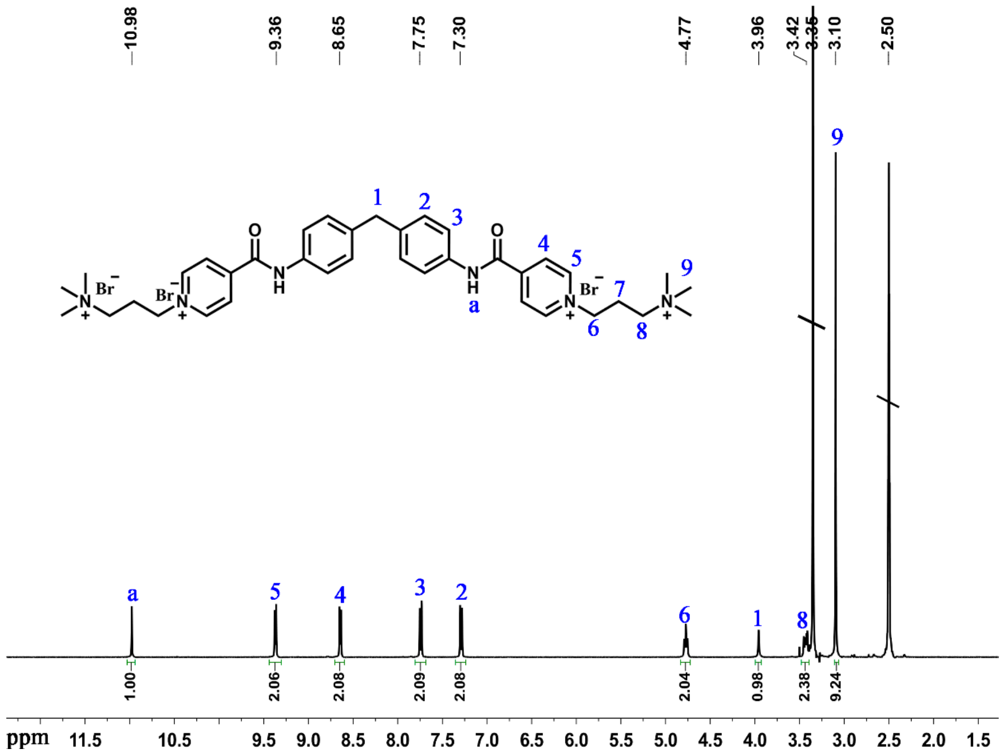

Figure 25. ^{1}H NMR spectrum (400 MHz, DMSO-d_6, 293 K) of compound **MD1.**

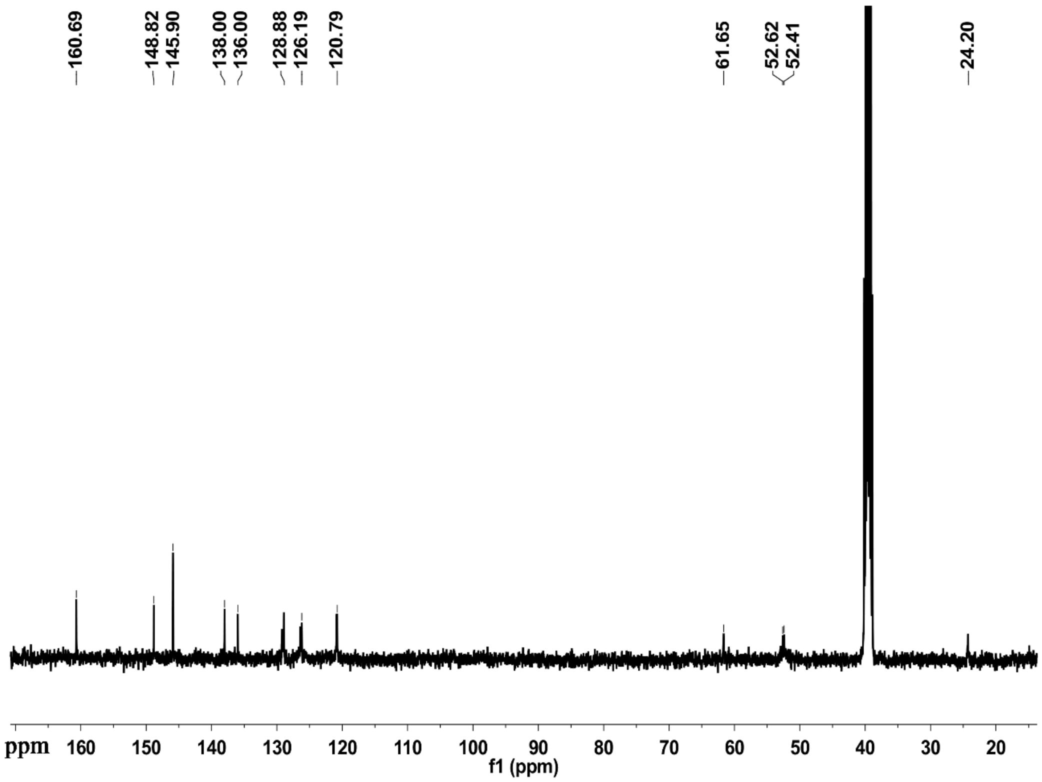

Figure S26. ^{13}C NMR spectrum (100 MHz, DMSO-d_6, 293 K) of compound **MD1**.

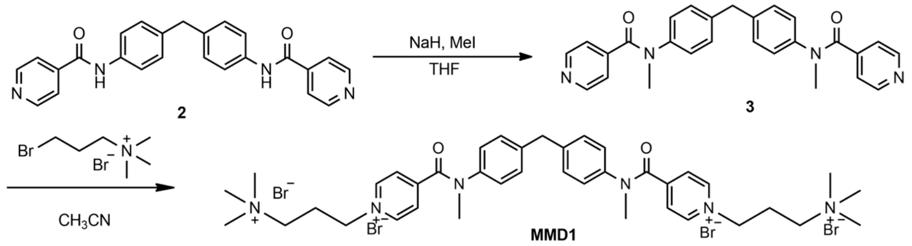

Scheme S8. Synthesis of **MMD1**.

Sodium hydride (0.027 g, 0.60 mmol) was added into the THF solution containing compound **2** (0.045 g, 0.10 mmol) and the mixture solution was stirred for 5 h under 50 °C. Then, 2 mL THF of Methyl iodide (0.094 g, 0.60 mmol) was added into the mixture solution and reflux for 4 h. Then, water and ethyl acetate were successively added to the resulting mixture. The organic phase was separated and dried to afford compound **3**. **3** (0.024 g, 0.056 mmol) was dissolved in 5 mL of DMF and the solution was added dropwise into a refluxing CH_3CN solution (50 mL) of 3-bromo-N, N, N-trimethylolpropane-1-aminium bromide (0.12 g, 0.45 mmol). The mixture was refluxed for 8 hours, and the precipitate was filtered off, washed several times with CH_3CN, diethyl ether, then dried over vacuum get pure **MMD1** (12 mg, 23%). ^{1}H NMR (400 MHz, DMSO-d_6, ppm):δ 9.05 (d, J = 0.9, 2H), 8.06 (d, J = 4.9, 2H), 7.27 (d, J = 8.0, 2H), 7.17 – 7.07 (m, 2H), 4.59 (t, 2H), 3.78 (s, 1H), 3.34 (m, 2), 3.08 (s, 3H), 3.07 (s, 9H), 2.37 (m, 2H). ^{13}C NMR (100 MHz, DMSO-d_6, ppm): δ 164.9 (CO), 152.6 (CH), 145.8 (CH), 140.8 (CH),145.5 (CH), 135.8 (CH), 128.3 (CH), 126.7 (CH), 65.4 (CH), 62.1 (CH), 58.0 (CH), 53.0 (CH), 37.9 (CH), 26.0 (CH), 24.7 (CH).

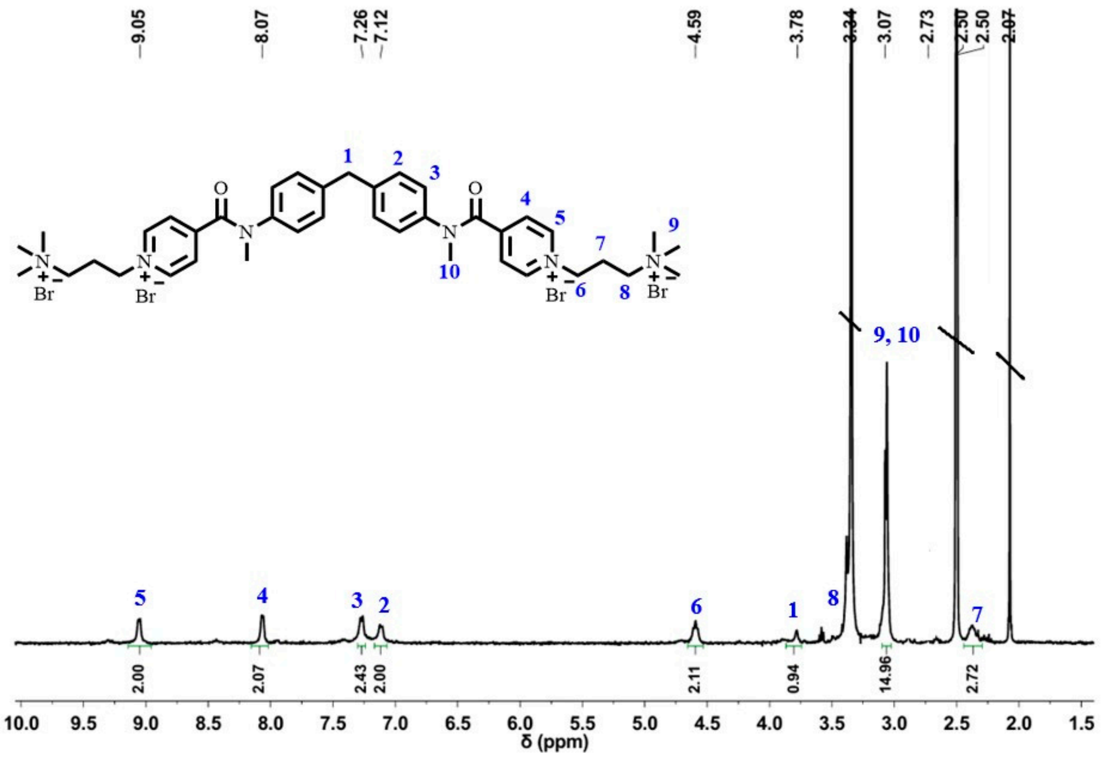

Figure 27. ^{1}H NMR spectrum (400 MHz, DMSO-d_6, 293 K) of compound **MMD1.**

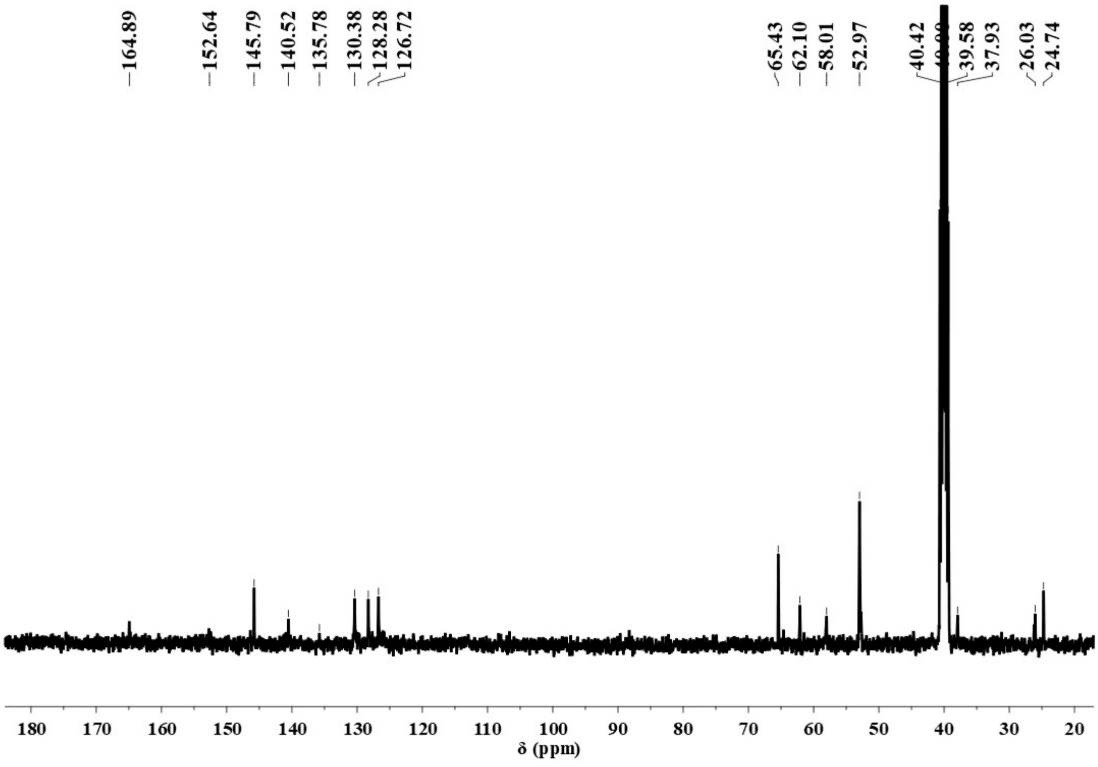

Figure S28. ^{13}C NMR spectrum (100 MHz, DMSO-d_6, 293 K) of compound **MMD1**.

2. Bacteria Killing Studies

Table S1. *In vitro* antibacterial activity of compounds **M1–M3**, **D1A–D3A,** and polymyxin B.

Compound	Bacterial strain			
	***E. coli* (ATCC 25922)**		***S. aureus* (ATCC 25923)**	
	**MIC** (μM)	**MBC** (μM)	**MIC** (μM)	**MBC** (μM)
**M1**	>15000	>15000	>15000	>15000
**M2**	>15000	>15000	>15000	>15000
**M3**	>15000	>15000	>15000	>15000
**D1A**	>15000	>15000	>15000	>15000
**D2A**	>15000	>15000	>15000	>15000
**D3A**	>960	>960	>960	>960
Polymyxin B	1.88	3.75	60	120

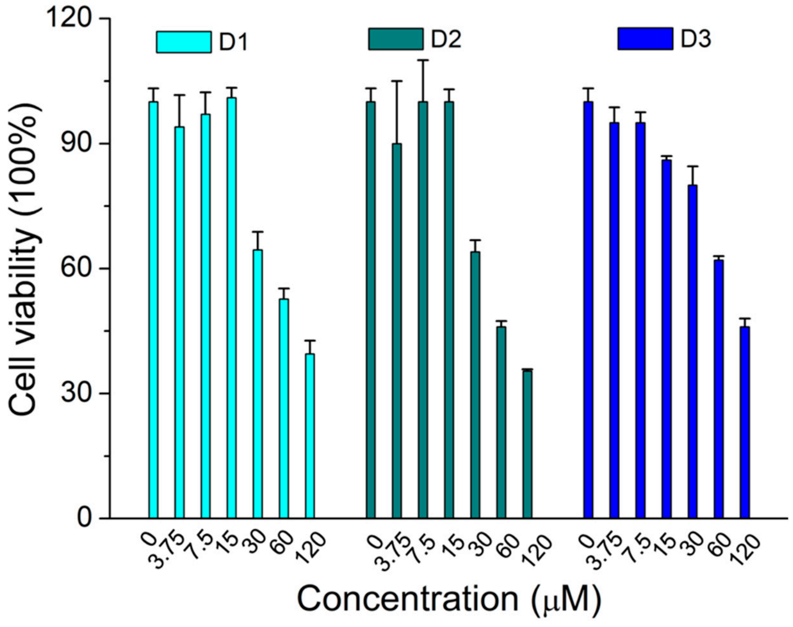

Figure S29. *In vitro,* cytotoxicity of **D1**, **D2**, and **D3** was determined by CCK8 assay against L929 cells after 24 h incubation. PBS (pH = 7.4) was employed as negative Control. Data are presented as the mean ± SD, n = 3.

Table S2. *In vitro* antibacterial activity of compounds **MD1** and **MMD1**.

	Bacterial strains									
	E. coli		*P. aeruginosa*		*S. aureus*		*S. epidermidis*		*E. faecalis*	
	MIC	MBC	MIC	MBC	MIC	MBC	MIC	MBC	MIC	MBC
**MD1**	960	>960	>960	>960	960	>960	480	>960	240	480
**MMD1**	>960	>960	>960	>960	>960	>960	>960	>960	960	>960

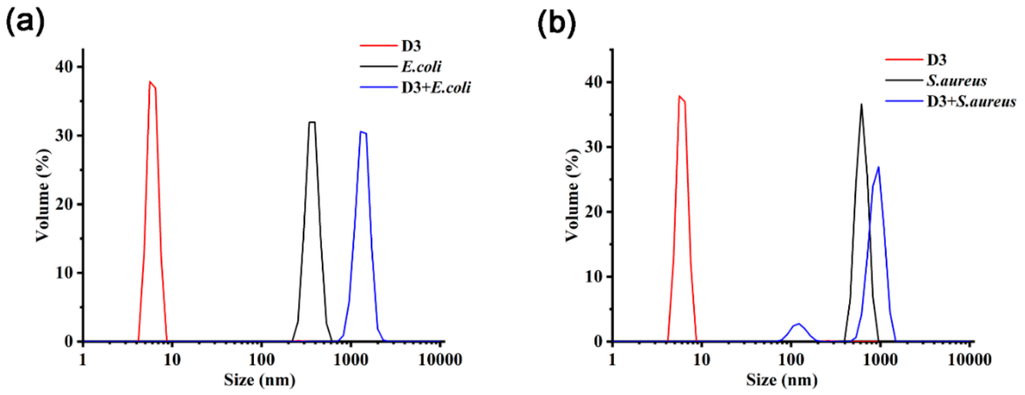

Figure S30. DLS results of the bacterial suspensions of (a) *E. coli* and (b) *S. aureus* before and after the addition of **D3** for 60 min.

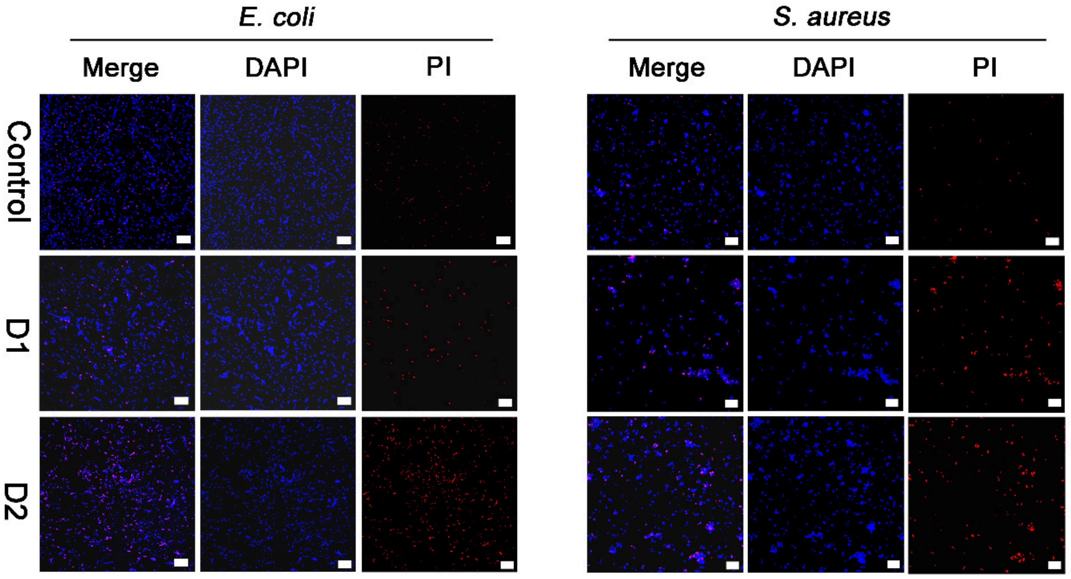

Figure S31. CLSM images of *E. coli* and *S. aureus* after treatment with control (PBS), and urea-based receptors **D1**, and **D2**, respectively. From left to right, the images in columns show merged images, blue signals from DAPI, and red signals from PI.and merged images (scales bar: 20 μm)

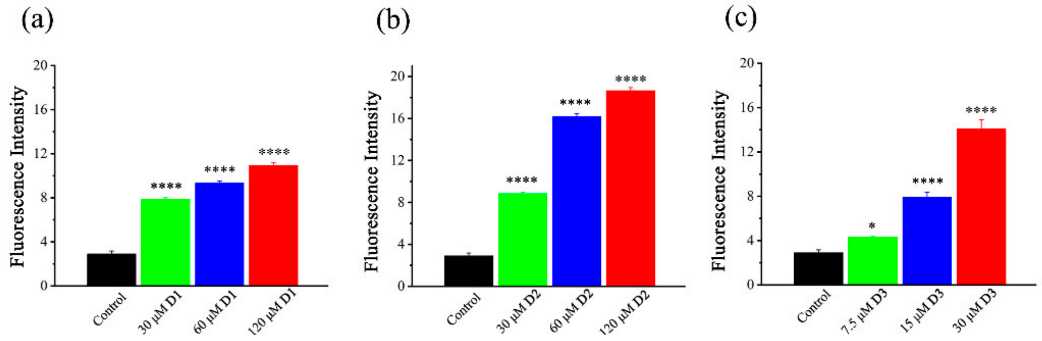

Figure S32. Cell membrane permeability of *E. coli* treated with various receptors at different concentrations after 120 min (a) **D1**, (b) **D2**, (C) **D3**, using propidium iodide (PI) as the dye (em = 617 nm). Data are presented as the mean ± SD, n = 3. *$p < 0.05$, ****$p < 0.0001$.

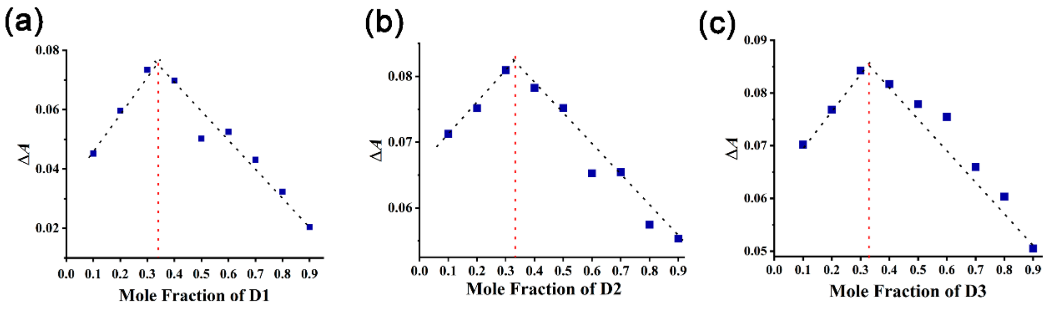

Figure S33. (a-c) **D1**, **D2**, and **D3** and dibutyl phosphate in DMSO/$CHCl_3$. (1%, v/v, DMSO was added in the cases of **D1** (or **D2**, **D3**) to ensure a good solubility). The observed maximum at molar fraction of 0.33 is consistent with the proposed 1:2 complex between compounds **D1**, **D2**, **D3,** and dibutyl phosphate; [**D1**] + [Dibutyl Phosphate] = [**D2**] + [Dibutyl Phosphate] = [**D3**] + [Dibutyl Phosphate] = 1.0×10^{-5} M.

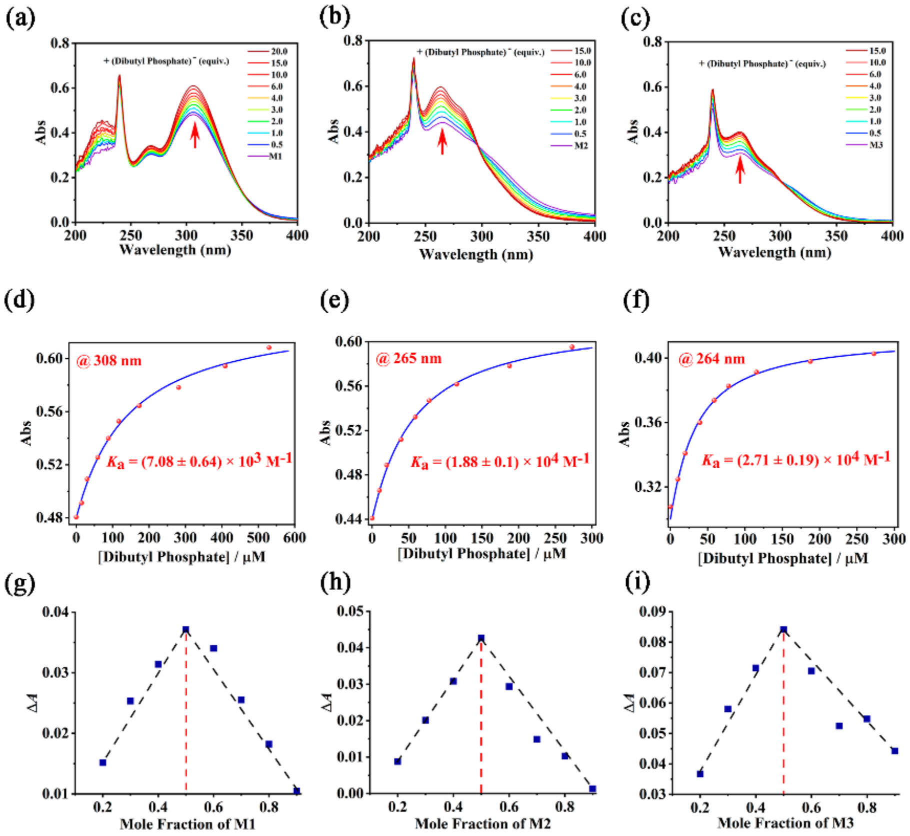

Figure S34. (a-c) UV titration spectra for determining the binding constants of (a) **M1** (30 μM)**,** (b) **M2** (20 μM)**,** (c) **M3** (20 μM) for anion guests, (d-f) show the corresponding association constants (K_a) determined by fitting the titration data (red dots) at the wavelength as noted to a 1:1 (host : guest) binding model (blue solid line) by the Dynafit program[1]. (g-i) Job Plots were carried out between (g) **M1**, (h) **M2**, and (i) **M3** and dibutyl phosphate in DMSO/$CHCl_3$. (1%, v/v, DMSO was added in the cases of **M1** (or **M2**, **M3**) to ensure a good solubility). The observed maximum at molar fraction of 0.5 is consistent with the proposed 1:1 complex between compounds **M1**, **M2**, **M3,** and dibutyl phosphate; [**M1**] + [Dibutyl Phosphate] = [**M2**] + [Dibutyl Phosphate] = [**M3**] + [Dibutyl Phosphate] = 5.0×10^{-5} M.

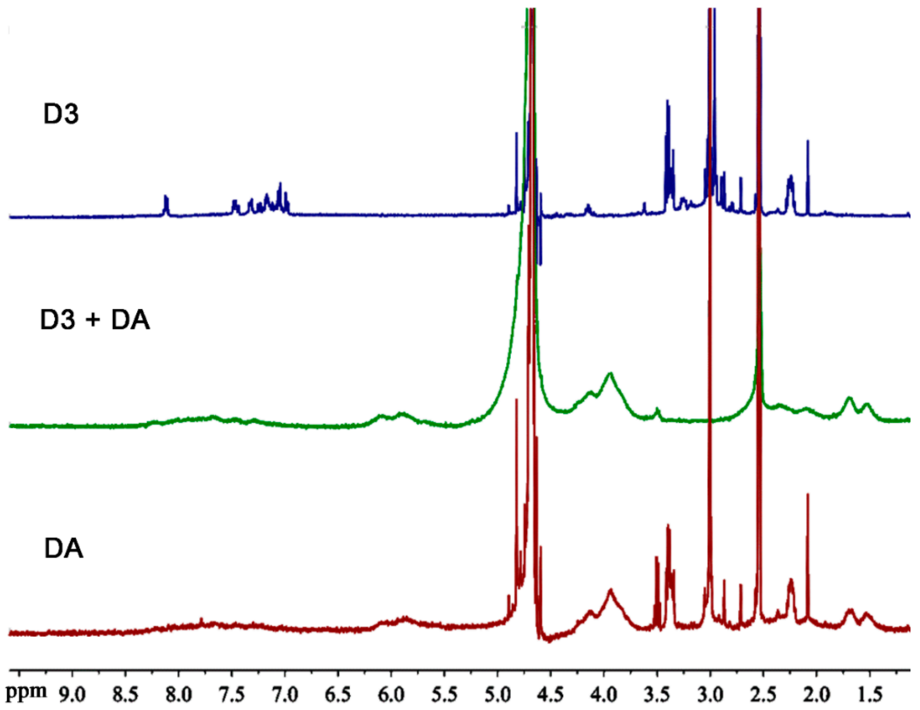

Figure S35. ^{1}H NMR spectra of **D3**, DA, and the mixture of **D3** and DA (400 MHz, D_2O/DMSO-d_6 (9/1, *v*/*v*), 293 K).

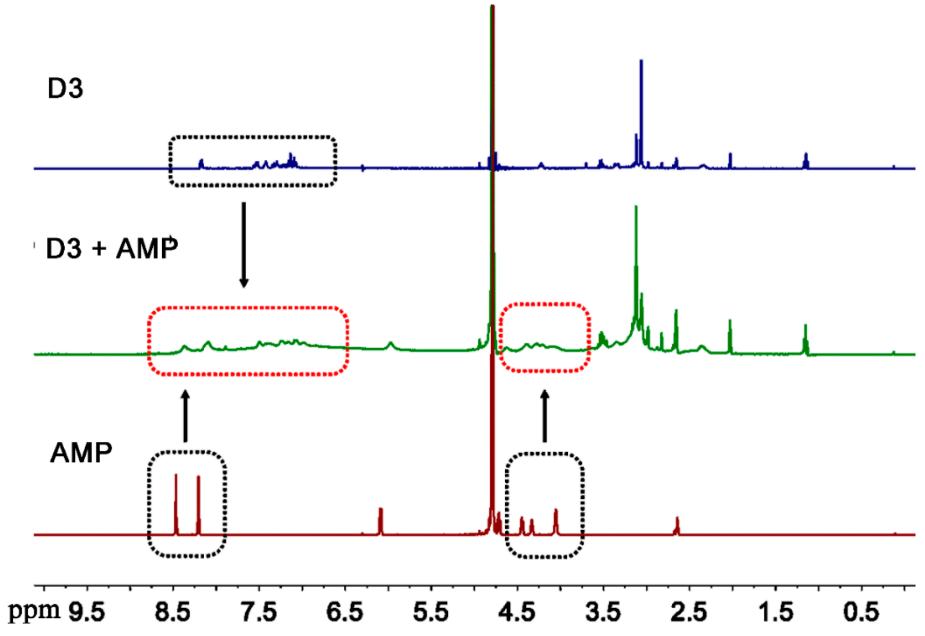

Figure S36. ^{1}H NMR spectra of **D3**, AMP, and the mixture of **D3** and AMP (400 MHz, D_2O/DMSO-d_6 (9/1, *v*/*v*,), 293 K).

Table S3 The quenching constants (K_q) of receptors with CT-DNA.

Compound	K_q [EB-(CT-DNA)]
**D1**	9.85×10^5 M^{-1}
**D2**	1.75×10^6 M^{-1}
**D3**	7.88×10^6 M^{-1}
**M1**	1.36×10^4 M^{-1}
**M2**	2.59×10^4 M^{-1}
**M3**	1.05×10^5 M^{-1}

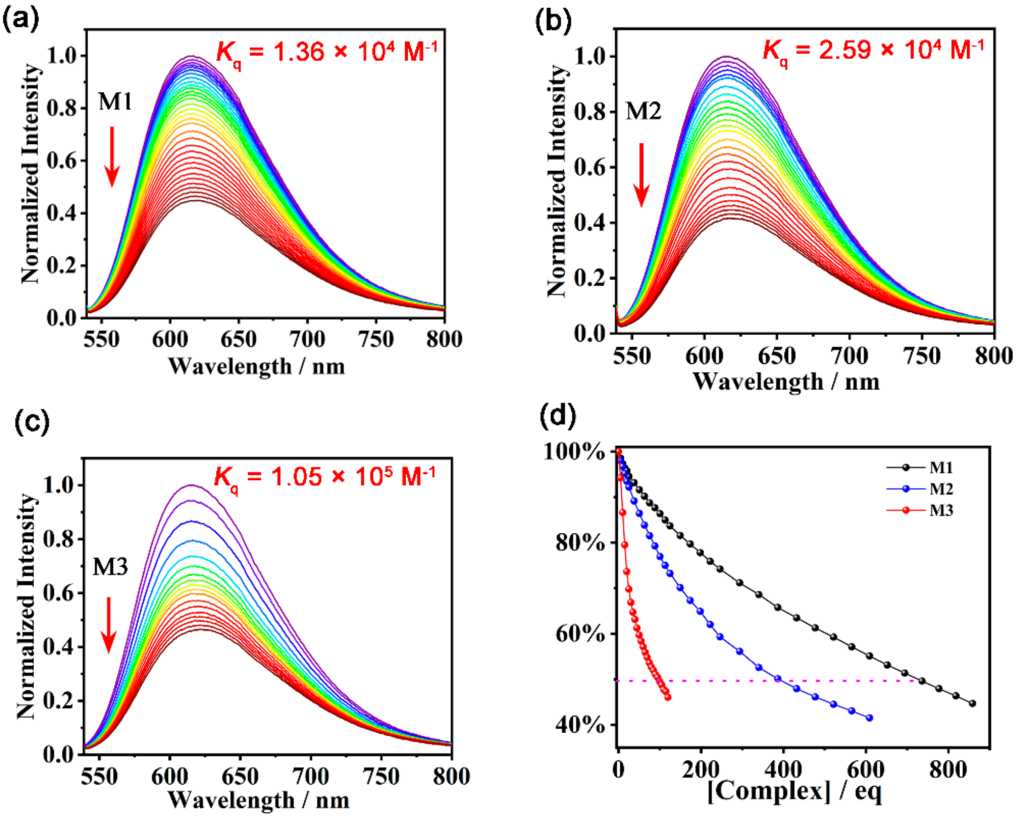

Figure S37. Fluorescence spectra of ethidium bromide (EB, 1.3 μM) bound to calf thymus DNA (CT-DNA, 3.8 μM) (buffer: 5 mM Tris-HCl/50 mM NaCl, pH = 7.26) before and after addition of aliquots of (a)**M1**, (b) **M2** and (c) **M3**. (d) The percentage decrease in fluorescence with the addition of **M1**, **M2** and **M3**, respectively.

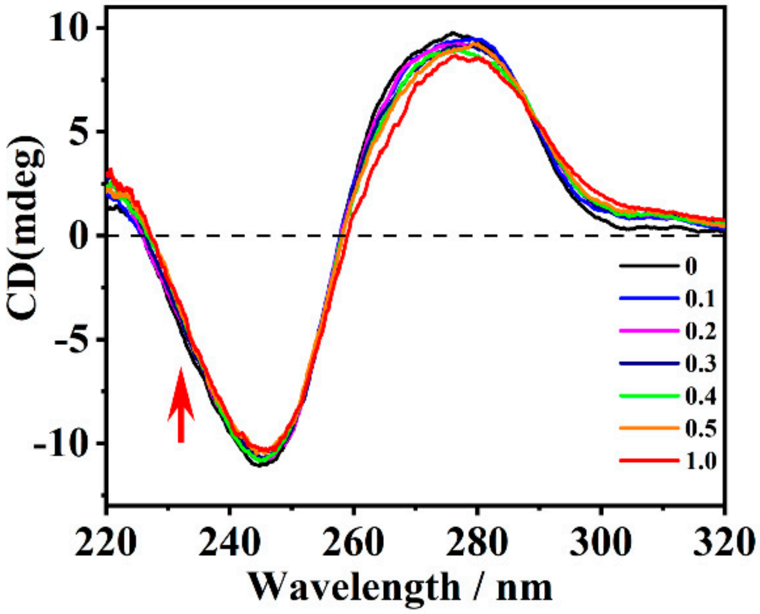

Figure S38. CD spectra of CT-DNA (0.1 mM) with the addition of different molar ratios of **M3**.

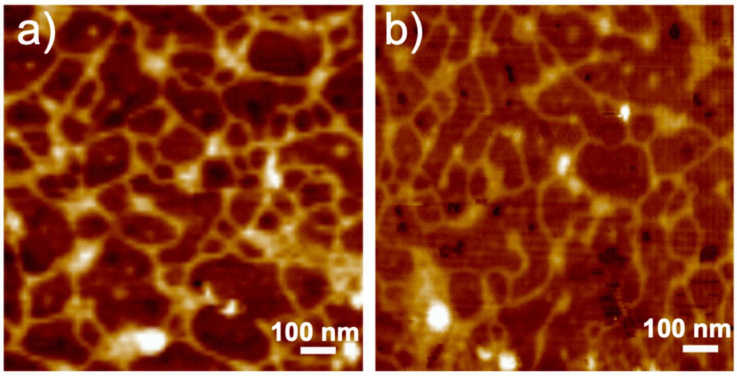

Figure S39. AFM images of pDNA before (a) and after the addition of **D1** with the concentration of 15 μM (b).

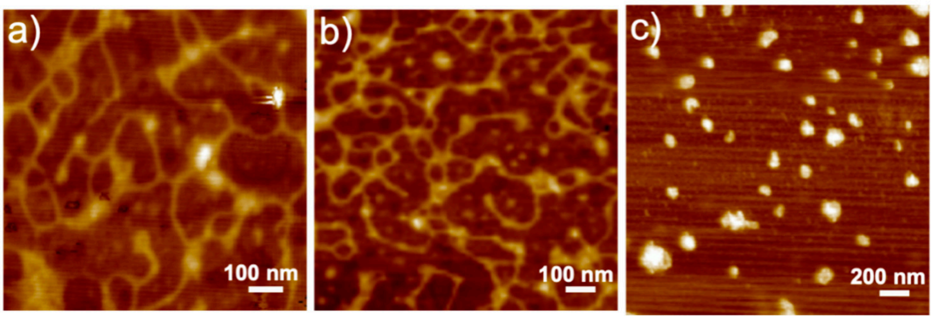

Figure S40. AFM images of pDNA before (a) and after the addition of **D2** with the concentration of 15 μM (b) and 60 μM (c).

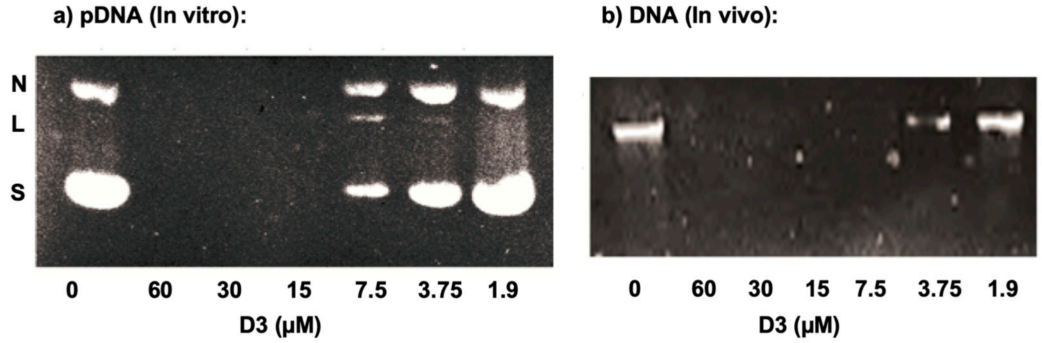

Figure S41. (a) Agarose gel electrophoresis retardation assay pDNA (*in vitro*) treated with **D3**, and extracted DNA in *E. coli* cells treated with **D3** for 12 h(concentrations as labelled in μM, labels for pDNA and DNA, N = nicked, L = linear, and S = supercoiled).

3. Reference

[1] P. Kuzmic, Program DYNAFIT for the analysis of enzyme kinetic data: application to HIV proteinase [J]. Anal. Biochem., **1996**, 237, 260-273.
